# A conserved acetylation switch enables pharmacological control of tubby-like protein stability

**DOI:** 10.1074/jbc.RA120.015839

**Published:** 2020-11-23

**Authors:** Evan M. Kerek, Kevin H. Yoon, Shu Y. Luo, Jerry Chen, Robert Valencia, Olivier Julien, Andrew J. Waskiewicz, Basil P. Hubbard

**Affiliations:** 1 Department of Pharmacology, University of Alberta, Edmonton, Alberta, Canada; 2 Department of Biological Sciences, University of Alberta, Edmonton, Alberta, Canada; 3 Department of Biochemistry, University of Alberta, Edmonton, Alberta, Canada

**Keywords:** tubby-like proteins (TULPs), TULP3, posttranslational modification (PTM), acetylation, histone acetyltransferases (HATs), E1A binding protein p300 (p300), histone deacetylases (HDACs), histone deacetylase 1 (HDAC1), E3 ubiquitin ligase, Cullin-3, TULP1, TULP2, TULP4, ATCC, American Type Culture Collection, co-IP, coimmunoprecipitation, GPCRs, G-protein-coupled receptors, HATs, histone acetyltransferases, HDACs, histone deacetylases, IFT, intraflagellar transport, IP-MS, immunoprecipitation–mass spectrometry, MSCV, murine stem cell virus, PKD, polycystic kidney disease, PTM, posttranslational modification, SOCS, suppressor of cytokine signaling, TSA, trichostatin A, TULPs, tubby-like proteins

## Abstract

Tubby-like proteins (TULPs) are characterized by a conserved C-terminal domain that binds phosphoinositides. Collectively, mammalian TULP1-4 proteins play essential roles in intracellular transport, cell differentiation, signaling, and motility. Yet, little is known about how the function of these proteins is regulated in cells. Here, we present the protein–protein interaction network of TULP3, a protein that is responsible for the trafficking of G-protein-coupled receptors to cilia and whose aberrant expression is associated with severe developmental disorders and polycystic kidney disease. We identify several protein interaction nodes linked to TULP3 that include enzymes involved in acetylation and ubiquitination. We show that acetylation of two key lysine residues on TULP3 by p300 increases TULP3 protein abundance and that deacetylation of these sites by HDAC1 decreases protein levels. Furthermore, we show that one of these sites is ubiquitinated in the absence of acetylation and that acetylation inversely correlates with ubiquitination of TULP3. This mechanism is evidently conserved across species and is active in zebrafish during development. Finally, we identify this same regulatory module in TULP1, TULP2, and TULP4 and demonstrate that the stability of these proteins is similarly modulated by an acetylation switch. This study unveils a signaling pathway that links nuclear enzymes to ciliary membrane receptors via TULP3, describes a dynamic mechanism for the regulation of all tubby-like proteins, and explores how to exploit it pharmacologically using drugs.

The *tubby* phenotype, characterized by mature-onset obesity, insulin resistance, sterility, and hearing and vision impairment, was first observed in an inbred strain of C57BL/6J mice (1, 2, 3, 4). Using positional cloning, the cause of these pathologies was later determined to be a splicing defect in the eponymous tubby (TUB) gene (5). TUB and its related tubby-like proteins (TULPs) comprise a family of proteins present in both plant and animal kingdoms that are distinguished by a highly conserved C-terminal domain (6). This domain consists of a central alpha helix enclosed by a beta barrel structure and includes two amino acids (Lys330 and Arg332) that coordinate binding to membrane phosphoinositides such as phosphatidylinositol 4,5,-bisphosphate (PIP2) through an electrostatic interaction (7). The N-terminal regions of TUB and TULP homologs are variable and may be divided into three distinct classes: (1) those containing WD40 domains and/or suppressor of cytokine signaling (SOCS) motifs, (2) those without any apparent domains/motifs, and (3) those containing F-box domains (8). TUB/TULPs carry out a diverse range of important physiological functions ranging from biotic and abiotic stress defense in plants to regulation of cell metabolism, intracellular transport, and neural differentiation in animals (8). Mutations in these genes have been associated with numerous conditions including impaired development (9), altered life span (10), kidney disease (11, 12), and several cancers (13, 14).

Four TULPs (TULP1-4) displaying unique tissue distribution, intracellular localization, and function have been described in mammals (4). TULP1 and TULP2 display selective expression in the retina and testis, respectively (15). In contrast, both TULP3 and TULP4 show widespread expression throughout development and in adulthood (16, 17). TULP3 is distributed equally in nuclear and cytoplasmic compartments, while TULP1 is localized to the inner segment of photoreceptors, and TULP2–TULP4 are predominately cytoplasmic (4, 18). Mutations in TULP1 result in progressive photoreceptor degeneration in mice and retinitis pigmentosa in humans (4, 19). The severity of this phenotype is further worsened in a *tubby* background (20). To date, TULP2 and TULP4 knockout mice have not been generated (4).

Whole-body knockout of TULP3 in mice causes polydactyly (21) and failed neural tube closure resulting in embryonic lethality (9). Furthermore, nephron-specific TULP3 knockout mice develop cystic kidneys (11, 12). These striking phenotypes have been attributed to improper trafficking of membrane proteins to cilia caused by TULP3 deletion (4). The N-terminus of TULP3 contains a conserved helix that interacts with the IFT-A complex, a component of intraflagellar transport (IFT) particles, which are required to assemble cilia and for trafficking inside cilia (4, 22, 23). With the IFT-A complex, TULP3 acts as an adaptor for the transport of at least 16 class A cilia-targeted G-protein-coupled receptors (GPCRs) including melanin-concentrating hormone receptor, neuropeptide Y receptors, and GPR161, a repressor of sonic hedgehog signaling (24). TULP3-IFT-A is also responsible for the ciliary transport of other integral membrane proteins such as the Polycistin 1/2 complex, which is implicated in polycystic kidney disease (PKD) (24). Three steps have been proposed for how this transport occurs: (1) capture of membrane cargo by TULP3 in a PIP2-dependent manner, (2) association with IFT-A and transport to cilia, and (3) release into a PIP2-deficient ciliary membrane (24). Overexpression of Inpp5e, an enzyme that hydrolyzes the 5ʹ phosphate from PIP2, has been shown to reduce TULP3 and GPR161 localization to cilia, indicating that low levels of PIP2 are required to anchor these proteins in place (25).

TULP3 contains a nuclear localization sequence that overlaps with its IFT-A binding region (4). Furthermore, it has been demonstrated that hydrolysis of PIP2 induces relocalization of TULP3 to the nucleus (8). While some evidence suggests that TULP3 and other TULPs might have the ability to directly bind DNA and act as transcription factors, their function in the nucleus is unclear (26). Moreover, little is known about how the localization, stability, and activity of tubby-like proteins might be regulated.

Here, we use Immunoprecipitation–Mass Spectrometry (IP-MS) to catalog the entire protein interaction network of TULP3. In addition to confirming previously validated interactions, we identify many new putative interactors, including enzymes such as SIRT1 and HDAC1 and Cullin-3, which regulate lysine acetylation (27, 28) and ubiquitination (29), respectively. We find that acetylation of TULP3 by p300 on several key residues including Lys316 and Lys389 increases its protein stability, while deacetylation by HDAC1 decreases TULP3 protein levels through a pathway involving proteasomal degradation. We use LC-MS/MS to map the ubiquitination sites on TULP3 and identify one site (Lys316) that can support either acetylation or ubiquitination and show that TULP3 ubiquitination correlates inversely with acetylation. We establish that this regulatory mechanism is active in zebrafish during embryonic development.

Highlighting the fundamental importance of this regulatory pathway, we find that some of these lysine residues are partially conserved and that Lys389 is fully conserved throughout all members of the tubby-like protein family in mammals. We show that TULP1, TULP2, and TULP4 protein levels are regulated by a parallel lysine acetylation switch. These data help define a new posttranslational regulatory pathway that facilitates rapid cross talk between chromatin modulating enzymes in the nucleus and membrane-bound receptors in cilia via TULP3. Additionally, through the application of small-molecule p300 and histone deacetylase (HDAC) inhibitors, this study presents a framework for future therapeutic applications by illustrating how the stability of all tubby-like proteins can be altered using drugs.

## Results

### TULP3 interacts with a diverse set of cytoplasmic and nuclear proteins

**Figure 1:**
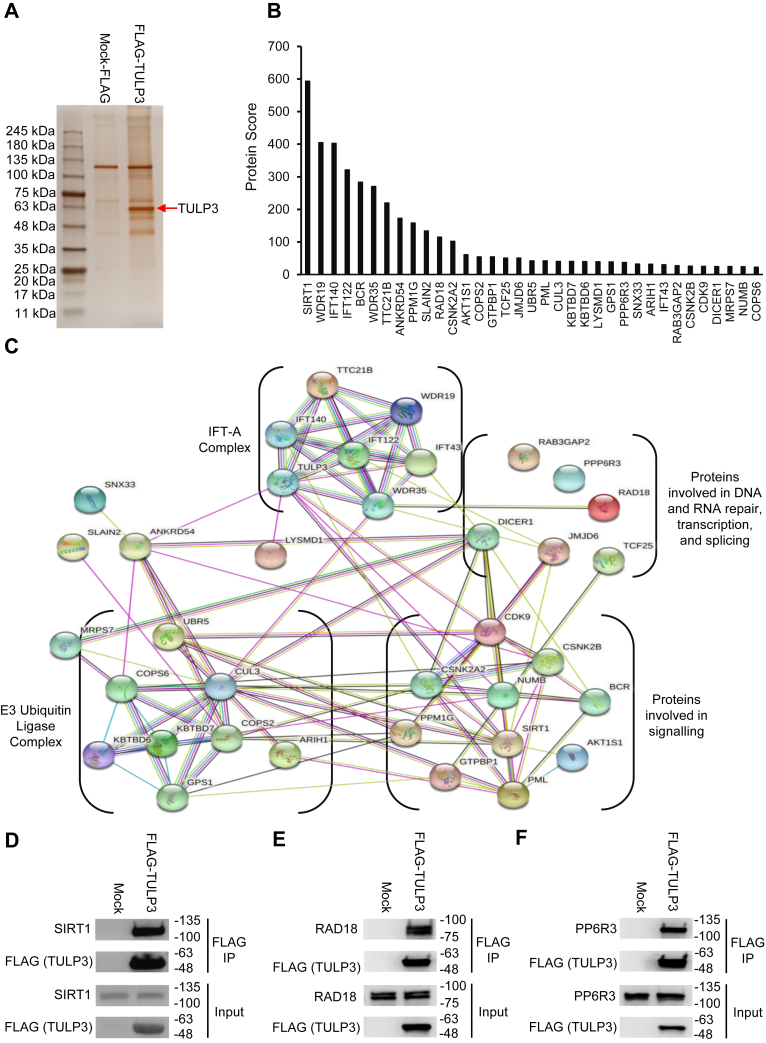
**Identification and validation of TULP3 protein–protein interactions.***A*, silver stained gel showing proteins coimmunoprecipitated alongside mock-FLAG or FLAG-TULP3 from 293T cells. The band corresponding to FLAG-TULP3 is indicated by a *red arrow*. *B*, bar graph ranking the top 35 high-confidence hits in (*A*) as identified by mass spectrometry. *C*, STRING diagram outlining the interconnectedness of TULP3 interactors identified in (*B*). Western blots showing coimmunoprecipitation of FLAG-TULP3 with (*D*) SIRT1, (*E*) RAD18, and (*F*) PP6R3 in 293T cells.

To better understand the biological role of TULP3 in the nucleus and to identify potential regulatory mechanisms for this protein, we performed an IP-MS experiment to profile its protein interactome. We transfected HEK293T cells with either mock-FLAG or FLAG-TULP3 and immunoprecipitated the resulting complexes using anti-FLAG coated beads (Fig. 1*A*). After identifying proteins in the IPs by mass spectrometry (Supplemental File 1; tabs 1,2), we generated a high-confidence list of TULP3 interactors by removing proteins present in the mock-FLAG IP as well as proteins having high average spectral counts of >2.1 in the CRAPome database (30) (Supplemental File 1; tabs 3,4). We used the top 35 of these 221 hits, ordered by Protein Score, to generate a STRING (31) network diagram (Fig. 1, *B*–*C*). This analysis revealed several nodes containing proteins involved in the IFT-A complex, nucleic acid repair, transcription, and splicing, mediators of cell signaling, and components of the Cullin-3 RING ubiquitin ligase complex (CRL-3) (32) (Fig. 1*C*). Of particular interest, we also identified interactions with several nuclear lysine deacetylase enzymes including SIRT1 and HDAC1 (27, 28, 33) (Fig. 1, *B*–*C*, Supplemental File 1). Subsequently, we validated the results from our mass spectrometry screen by performing coimmunoprecipitation (co-IP) followed by western blot. We confirmed that FLAG-TULP3 pulled down SIRT1 in reciprocal co-IP experiments (Fig. 1*D*, Fig. S1*A*). As well, we validated TULP3 interactions with two other potential regulatory enzymes, RAD18 (Fig. 1*E*), a ubiquitin ligase involved in DNA double-strand break repair (34), and PP6R3/SAPS3 (Fig. 1*F*), a regulatory subunit of protein phosphatase 6 (35).

### Acetylation of TULP3 by p300 increases its protein levels in cells

**Figure 2:**
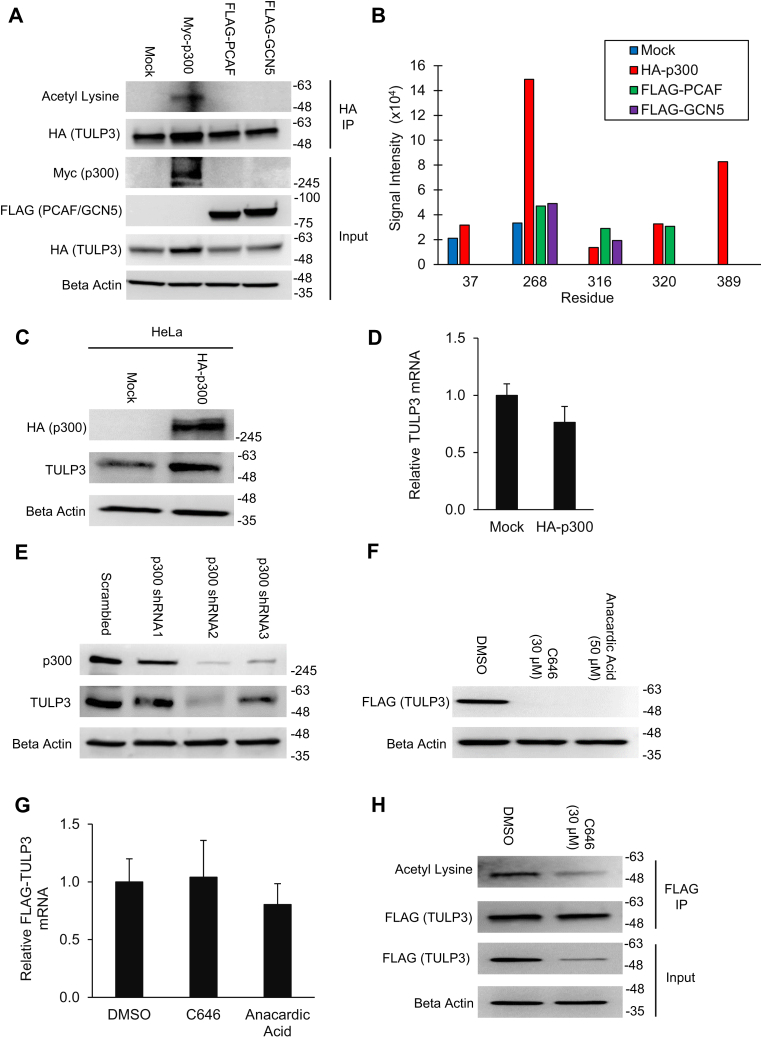
**Acetylation of TULP3 by p300 increases its protein abundance in cells**. *A*, western blot showing TULP3 acetylation levels in 293T cells in the presence of either empty pcDNA 3.1(+), Myc-p300, FLAG-PCAF, or FLAG-GCN5 following immunoprecipitation. Cells were harvested 48 h posttransfection. *B*, semiquantitative LC-MS/MS-based comparison of TULP3 acetylation corresponding to the conditions in (*A*). *C*, western blot showing total levels of endogenous TULP3 in HeLa cells following transfection with empty pcDNA 3.1(+) or a plasmid encoding HA-p300. Cells were harvested 48 h posttransfection. *D*, mRNA levels of endogenous TULP3 corresponding to samples in (*C*) examined using quantitative real-time PCR; n = 4 technical replicates, Mean ± S.D. shown. *E*, western blot of p300 and TULP3 protein levels following stable transduction of p300 shRNAs into HeLa cells. *F*, immunoblot of stably expressed FLAG-TULP3 protein in 293T cells following treatment with either DMSO, 30 μM C646, or 50 μM anacardic acid for 24 h. *G*, mRNA levels of FLAG-TULP3 corresponding to samples in (*F*) analyzed using quantitative real-time PCR; n = 4 technical replicates, Mean ± S.D. shown. *H*, acetylation levels of FLAG-TULP3 in stably expressing 293T cells following treatment with DMSO or 30 μM C646 for 9 h.

Based on our observations that TULP3 physically interacts with enzymes involved in lysine acetylation and ubiquitination, we hypothesized that TULP3 might be posttranslationally regulated by one or both of these modifications. To explore potential acetylation of TULP3, we performed an immunoprecipitation of HA-tagged TULP3 from 293T cells in the absence or presence of tagged histone acetyltransferase (HAT) enzymes including p300, PCAF, and GCN5 and probed the resulting western blot with a pan-acetyl antibody. We observed a strong acetylation signal resulting from p300 stimulation (Fig. 2*A*), but not the other HATs, which overlapped with the predicted molecular weight of TULP3. This effect was associated with an apparent increase in the total protein levels of TULP3 in the input of the experiment (Fig. 2*A*). Next, we used LC-MS/MS to validate that the observed acetylation signal was due to acetylation on TULP3. We identified a total of five acetylated lysine residues (Lys37, Lys268, Lys316, Lys320, Lys389) in the presence of p300, several of which were also present under basal conditions, or in the presence of exogenous PCAF or GCN5 (Fig. 2*B*). To further investigate the p300-dependent increase in TULP3 levels observed in Figure 2*A*, we expressed exogenous p300 in HeLa cells and performed a western blot to evaluate TULP3 protein expression. Consistent with our previous result, TULP3 protein levels were increased by nearly twofold following p300 stimulation (Fig. 2*C*, Fig. S1*B*). Importantly, TULP3 mRNA expression was unchanged by p300, suggesting that the increase in protein levels was not due to increased transcription of the gene (Fig. 2*D*). We next examined the effect of knocking down p300 in HeLa cells on TULP3 protein levels. Stable knockdown of endogenous p300 protein resulted in a reduction in TULP3 protein levels (Fig. 2*E*, Fig. S1*C*). To distinguish if inhibition of p300 enzymatic activity *versus* complete loss of protein was sufficient to replicate these results, we treated cells with 30 μM of C646 or 50 μM anacardic acid, two potent small-molecule p300 inhibitors (36). Consistent with our knockdown results, we found that pharmacological inhibition of p300 HAT activity resulted in a reduction in TULP3 protein levels in excess of fivefold (Fig. 2*F*). This effect was not due to reduced TULP3 mRNA expression (Fig. 2*G*). To investigate if this effect was associated with a decrease in TULP3 acetylation, we performed an immunoprecipitation of FLAG-TULP3 from cells treated with either DMSO or C646, normalized protein levels between the two IPs, and probed with a pan-acetyl antibody. Indeed, drug treatment resulted in a decrease in total TULP3 acetylation, concomitant with reduced protein levels in the input (Fig. 2*H*). Finally, we confirmed a protein–protein interaction between TULP3 and p300 in cells by performing reciprocal co-IP experiments (Fig. S1, *D–E*) and demonstrated the ability of p300 to directly acetylate TULP3 using an *in vitro* assay (Fig. S1*F*). Overall, these results demonstrate that TULP3 is acetylated in a p300-dependent manner and that its hyperacetylation is associated with an increase in its protein abundance.

### Mutation of Lys316, Lys320, and Lys389 each increases TULP3 stability

**Figure 3:**
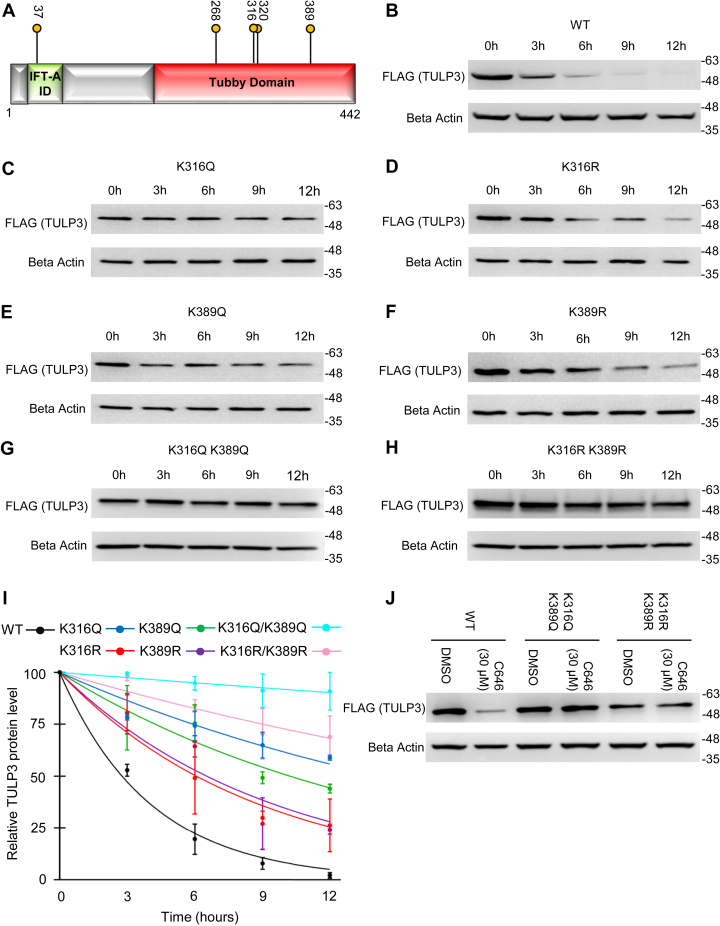
**Modification of K316, K320, and K389 each influences TULP3 stability.***A*, schematic outlining key functional domains in TULP3 and the location of acetylation sites. IFT-A ID denotes Intraflagellar Transport Complex A interacting domain. Western blots representing cycloheximide pulse-chase experiments for stably expressed (*B*) FLAG-TULP3 wild-type protein or (*C*) K316Q, (*D*) K316R, (*E*) K389Q, (*F*) K389R, (*G*) K316Q/K389Q, (*H*) K316R/K389R mutants in 293T cells. *I*, plot corresponding to experiments in (*B–H*) quantifying relative TULP3 protein levels *versus* time; n = 3 biological replicates, Mean ± S.D. shown. *J*, protein levels of FLAG-TULP3, FLAG-TULP3 K316Q/K389Q, and K316R/K389R mutants stably expressed in 293T cells following treatment with DMSO or 30 μM C646 for 9 h.

Previous studies have demonstrated a strong link between lysine acetylation and protein stability, often in the context of lysine acetylation/ubiquitination switches (37). Of the five acetylation sites we identified, four are located within the conserved tubby domain, and one is located within the N-terminal IFT-A interaction domain (Fig. 3*A*). We hypothesized that acetylation at one or more of these residues might directly affect TULP3 protein stability. To examine this possibility, we generated stable cells lines expressing Lys->Gln (acetyl mimetic) and Lys->Arg (deacetyl mimetic) mutant proteins (27) corresponding to each residue using site-directed mutagenesis. All of these proteins displayed nuclear-cytoplasmic localization patterns similar to the wild-type protein (Fig. S2). Next, we compared their stabilities to the wild-type protein by performing cycloheximide pulse-chase experiments. While the half-life of the wild-type protein was <3 h (Fig. 3*B*), we found that mutation of Lys316 or Lys389 to Gln or Arg greatly increased this (Fig. 3, *C*–*F*). Substitutions at other sites appeared to have little effect with the exception of K320Q, which also increased protein half-life (Fig. S3). Dual substitution of Lys316/Lys389 with Q or R increased TULP3 half-life by >20- and >7-fold, respectively, blocking virtually all degradation throughout the 12-h time window tested (Fig. 3, *G–I*). Moreover, these proteins were completely resistant to C646-induced TULP3 degradation (Fig. 3*J*). These data suggest that the mechanism by which p300 regulates TULP3 stability involves modification of key residues including Lys316 and Lys389.

### HDAC1 deacetylates TULP3 and reduces its protein levels

**Figure 4:**
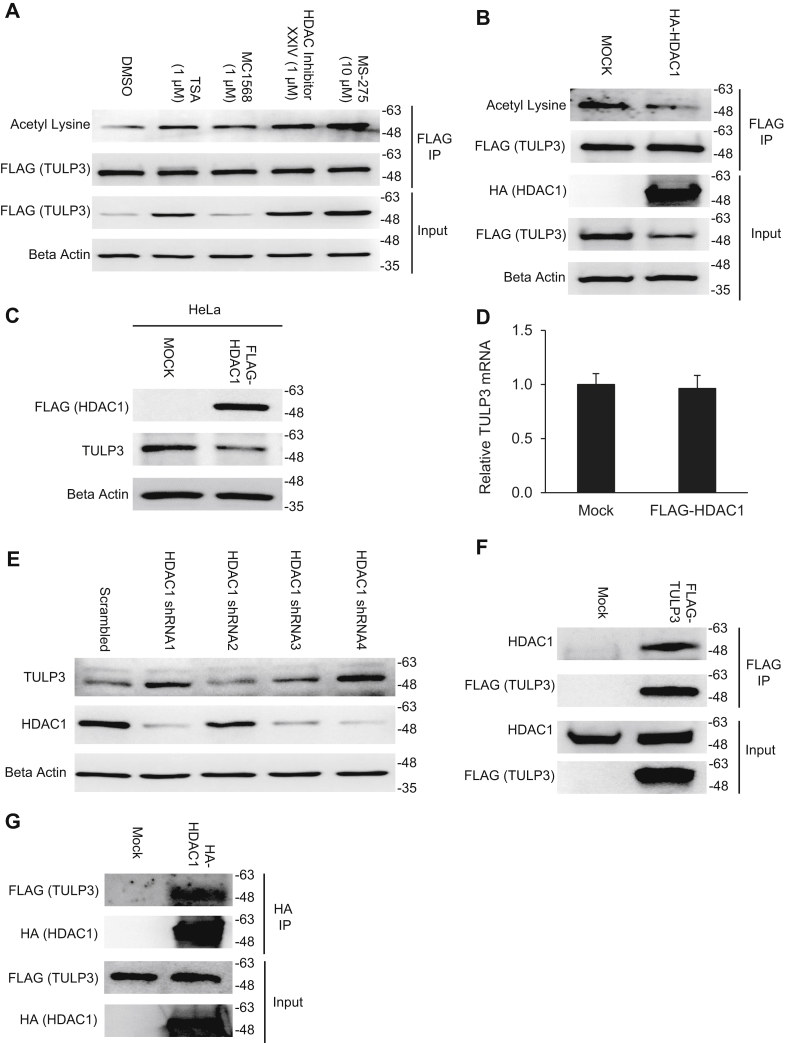
**HDAC1 deacetylates TULP3 and reduces its protein levels.***A*, western blot showing acetylation and total protein levels of stably expressed FLAG-TULP3 in 293T cells treated with DMSO, 1 μM TSA, 1 μM MC1568, 1 μM HDAC Inhibitor XXIV, or 10 μM MS-275 for 16 h. *B*, acetylation and total protein levels of stably expressed FLAG-TULP3 in 293T cells transfected with empty pcDNA 3.1(+) or a plasmid encoding HA-HDAC1. Cells were harvested 48 h posttransfection. *C*, western blot showing endogenous levels of TULP3 in Hela cells transfected with either empty pcDNA 3.1(+) or a plasmid encoding FLAG-HDAC1. Cells were harvested 48 h posttransfection. *D*, mRNA levels of endogenous TULP3 corresponding to treatments in (*C*) analyzed by qRT-PCR; n = 4 technical replicates, Mean ± S.D. shown. *E*, western blot of HDAC1 and TULP3 protein levels following stable transduction of HDAC1 shRNAs into HeLa cells. Western blots showing coimmunoprecipitation of *F* FLAG-TULP3 with HDAC1 or *G* HA-HDAC1 with FLAG-TULP3 in 293T cells.

Based on the results of the protein interaction screen (Fig. 1, Supplemental File 1), we reasoned that SIRT1 and HDAC1 were likely deacetylase candidates for TULP3. To test this assertion, we treated cells with various class I and class II HDAC as well as sirtuin inhibitors (38) and evaluated changes in FLAG-TULP3 acetylation (following normalization of protein levels) and total protein levels by immunoblot. We found that inhibition of SIRT1 using nicotinamide or EX-527 had no effect on TULP3 acetylation or protein levels (Fig. S4). However, treatment with several class I and class II HDAC inhibitors, namely trichostatin A (TSA), HDAC inhibitor XXIV, and MS-275, all boosted TULP3 protein levels by more than threefold and similarly affected protein acetylation levels (Fig. 4*A*). TSA is a pan-HDAC inhibitor while MS-275 selectively inhibits HDACs 1 and 3 and HDAC inhibitor XXIV inhibits numerous HDACs including HDAC1, but not HDAC3 (28). These results served to strengthen the hypothesis that HDAC1 might deacetylate TULP3 in cells. To confirm this, we expressed HA-HDAC1 in 293T cells and examined the effect on TULP3 protein levels and acetylation. We found that HDAC1 decreased TULP3 protein levels by >50% in addition to reducing its acetylation by an equal degree (Fig. 4*B*). We repeated this experiment in HeLa cells and found that HDAC1 expression lowered TULP3 protein without affecting its mRNA levels (Fig. 4, *C*–*D*). Next, we stably knocked down endogenous levels of HDAC1 in HeLa cells using lentivirus and examined the effect on TULP3 protein levels. We found that reduced levels of HDAC1 correlated with increased abundance of TULP3 (Fig. 4*E*), consistent with our hypothesis. We also validated the prospective TULP3–HDAC1 interaction that was initially identified by mass spectrometry (Supplemental File 1) by performing reciprocal co-IP experiments using FLAG-TULP3 and HA-HDAC1 (Fig. 4, *F*–*G*). These results identify HDAC1 as a primary TULP3 deacetylase.

### Acetylation of TULP3 blocks its ubiquitination

**Figure 5:**
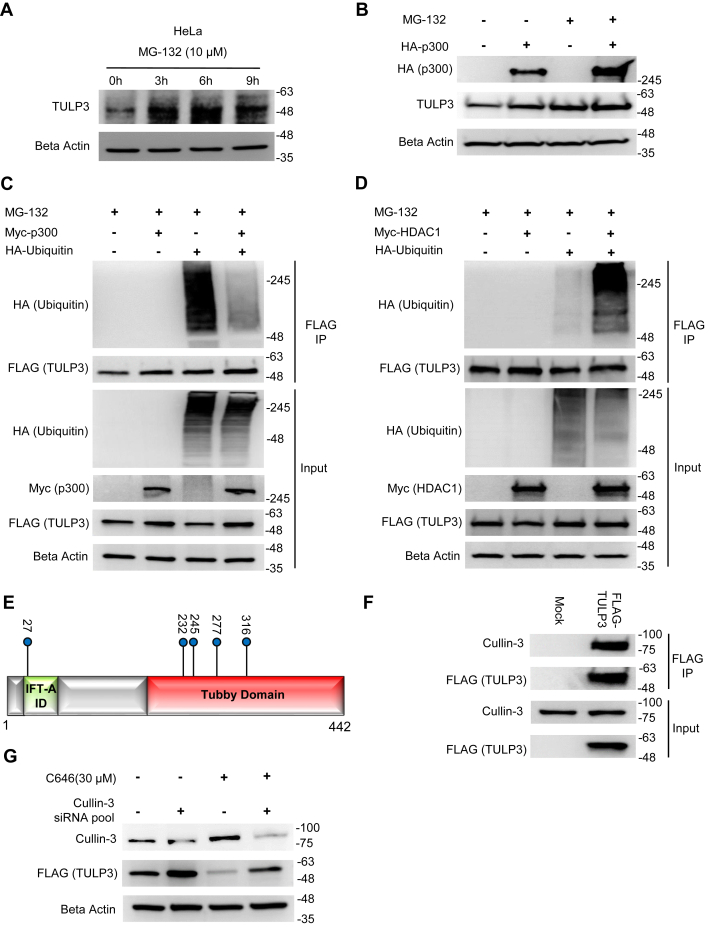
**TULP3 protein stability is regulated by polyubiquitination and by Cullin-3.***A*, western blot showing TULP3 levels in HeLa cells treated with 10 μM MG-132 for the time intervals indicated. *B*, TULP3 protein levels in the absence or presence of 10 μM MG-132 and transfected with a plasmid encoding HA-p300 as indicated. Cells were harvested 48 h posttransfection following 9 h of MG-132 treatment. *C*, immunoblot analysis of ubiquitination levels on FLAG-TULP3 immunoprecipitated from 293T cells treated with MG-132 and transfected with plasmids encoding HA-Ubiquitin or Myc-p300 as indicated. Cells were harvested 48 h posttransfection following 9 h of MG-132 treatment. *D*, western blot showing ubiquitination levels on FLAG-TULP3 immunoprecipitated from 293T cells treated with MG-132 and transfected with plasmids encoding HA-Ubiquitin or Myc-HDAC1 as indicated. Cells were harvested 48 h posttransfection following 9 h of MG-132 treatment. *E*, schematic outlining key functional domains in TULP3 and the location of ubiquitination sites identified by mass spectrometry. IFT-A ID denotes Intraflagellar Transport Complex A interacting domain. *F*, western blot showing coimmunoprecipitation of FLAG-TULP3 with Cullin-3 in 293T cells. *G*, immunoblot showing levels of FLAG-TULP3 protein in 293T cells in the absence or presence of C646 following transfection with a pool of siRNAs targeting Cullin-3 or a scrambled control. Cells were collected 72 h posttransfection; 30 μM C646 was added to cells 48 h posttransfection as indicated.

To build upon the observation that TULP3 interacts with Cullin-3 (Fig. 1) and to examine if polyubiquitination and subsequent proteasomal degradation might underlie the changes in protein stability caused by differential acetylation, we treated cells with the proteasome inhibitor MG-132, and observed its effect on expression of endogenous and exogenous TULP3. We found that MG-132 increased protein abundance of TULP3 over the course of a 9-h interval by approximately threefold without changing its mRNA levels (Fig. 5*A*, Fig. S5, *A–C*). Next, we set out to test whether the effects of p300 on TULP3 stability occur via this same mechanism or an independent mechanism. To do this, we expressed p300 in HeLa cells in the presence or absence of MG-132. While p300 and MG-132 each increased TULP3 protein individually, the effect was not additive, suggesting that both operate through a common pathway (Fig. 5*B*, Fig. S5*D*). Based on these results, we surmised that acetylation could be stabilizing TULP3 by blocking its ubiquitination. We immunoprecipitated FLAG-TULP3 cotransfected with HA-ubiquitin (and treated with MG-132) in the absence or presence of exogenous p300 and probed with an HA-antibody to detect ubiquitination. We found that p300 decreased TULP3 ubiquitination (Fig. 5*C*). To evaluate the corollary that TULP3 deacetylation increases its ubiquitination, we repeated with experiment with HDAC1 in place of p300. Consistent with our hypothesis, TULP3 ubiquitination levels were increased by expression of Myc-HDAC1 (Fig. 5*D*). These results were also reproduced using an antibody to endogenous ubiquitin (Fig. S5, *E–F*). To examine the interplay between acetylation at Lys316 and Lys389 and ubiquitination, we transfected cells with FLAG-tagged K316Q/K389Q and K316R/K389R TULP3 mutants alongside HA-ubiquitin in the presence of MG-132 and subsequently immunoprecipitated the mutant proteins and analyzed their ubiquitination levels by western blot. Compared with wild-type FLAG-TULP3, we found that both of the double acetylation mutant proteins displayed decreased levels of polyubiquitination, implicating these lysine residues in playing a role in TULP3 degradation (Fig. S5*G*). Next, we used LC-MS/MS to map ubiquitination sites on TULP3. We identified five ubiquitination sites (Fig. 5*E*), including Lys316, a target of p300-mediated acetylation and a mediator of TULP3 protein stability (Fig. 2 and Fig. 3). Finally, to test if the CRL-3 complex might be a cognate E3 ligase responsible for polyubiquitination of TULP3, we validated the putative interaction between TULP3 and Cullin-3 (Fig. 5*F*), and performed pooled siRNA knockdown of endogenous Cullin-3 to examine its effect on FLAG-TULP3 protein levels in cells. We found that knockdown of Cullin-3 increased TULP3 protein abundance by more than twofold and partially reversed the drop in TULP3 protein levels associated with C646 treatment (Fig. 5*G*). Collectively, these results demonstrate that an acetylation switch controls TULP3 protein stability and that p300, HDAC1, and the Cullin-3 RING ligase complex are involved in this mechanism.

### Deletion of the tubby domain in TULP3 renders the protein refractory toward C646 and MG-132 treatment

To fully map the determinants of the acetylation switch, we constructed a series of TULP3 truncation and deletion variants, spanning the N-terminal region (1–183) as well as the tubby domain (residues 184–442) (Fig. S6*A*). Initially, we performed nuclear-cytoplasmic cell fractionation with cells expressing the mutant proteins to establish their intracellular localization. Despite the removal of an NLS, we found that all TULP3 N-terminal deletion mutants remained distributed in both cytoplasmic and nuclear compartments (Fig. S6*B*). However, deletion of the tubby domain, which contains a second NLS sequence, resulted in complete redistribution of the protein to the cytoplasm (Fig. S6*B*). Unlike the wild-type protein or N-terminal deletion variants, we observed that the protein levels of these cytoplasmic, tubby-domain devoid TULP3 variants were unaltered by treatment with either C646 or MG-132 (Fig. S6, *C–D*). These data suggest that the residues responsible for controlling the stability of TULP3 via acetylation are contained within the tubby domain and/or that the enzymes mediating the effects of these drugs are sequestered in the nucleus.

### Acetylation regulates TULP3 protein stability in zebrafish

**Figure 6:**
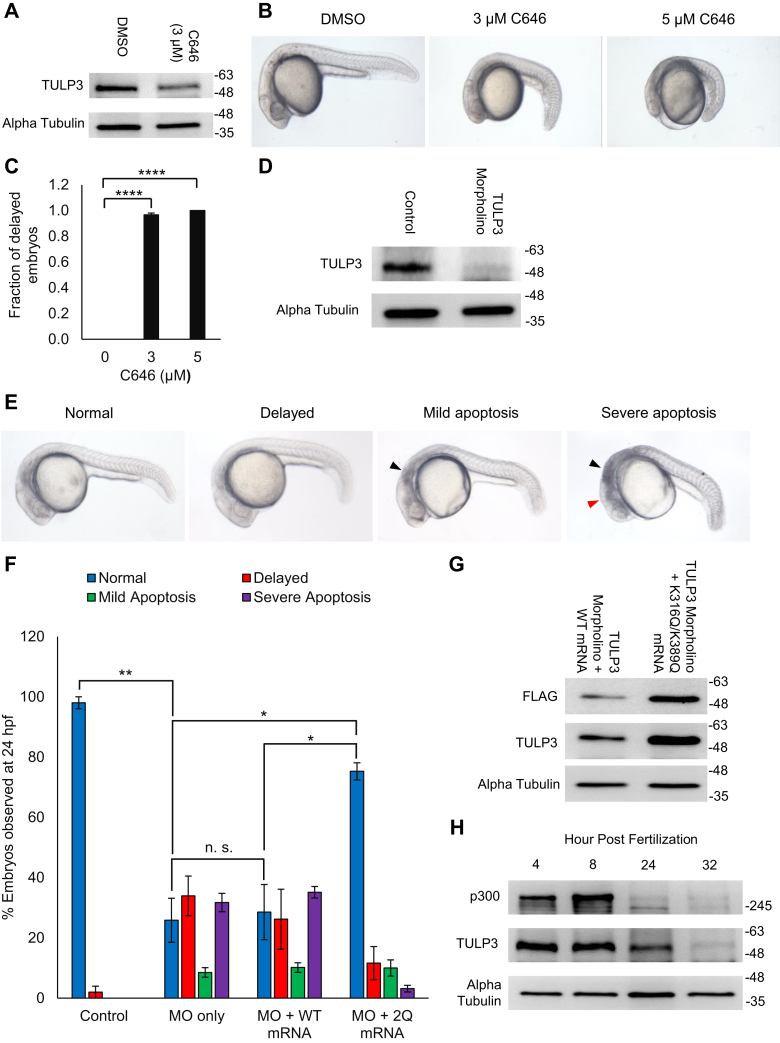
**An acetylation switch actively regulates TULP3 levels in zebrafish during development.***A*, immunoblot showing TULP3 protein levels in zebrafish embryo lysates harvested 10 h postfertilization following treatment with DMSO or 3 μM C646 for 10 h. *B*, representative photos of zebrafish embryos 24 h postfertilization treated with either DMSO or 3 or 5 μM C646 for 24 h. *C*, graph quantifying the frequency of the phenotypes observed in panel (*B*); n = 120, 123 or 123 for 0, 3, and 5 μM C646 groups respectively. Mean ± S.D. shown. ∗∗∗∗ denotes *p* < 0.0001 (*t* test). *D,* western blot showing levels of TULP in zebrafish embryos 24 h postfertilization in the absence or presence of 8 ng TULP3 morpholino. *E*, representative photos of zebrafish embryos 24 h postfertilization displaying normal, delayed, mild apoptosis, and severe apoptosis phenotypes. The *black* and *red arrows* indicate apoptosis in the hindbrain and forebrain, respectively. *F*, graph quantifying the frequency of the phenotypes observed in panel (*E*); n = 271, 248, 317, or 292 for control, morpholino only, morpholino + WT, or morpholino + 2Q mutant, respectively. Mean ± S.D. shown. ∗ denotes *p* < 0.05, ∗∗ denotes *p* < 0.01, and n.s. indicates nonsignificance (*t* test). *G*, levels of TULP3 protein in morpholino-injected zebrafish rescued with either wild-type TULP3 mRNA or K316Q/K389Q TULP3 mRNA. Lysates were harvested 8 h postfertilization. *H*, immunoblot showing protein levels of TULP3 and p300 in zebrafish embryo lysates harvested at 4, 8, 24, and 32 h postfertilization.

A sequence alignment of TULP3 orthologs in a variety of species revealed complete conservation of Lys316 and Lys389 (Fig. S7*A*). Since TULP3 plays a critical role in development (9), we chose to examine the physiological activity of the acetylation switch in zebrafish, an established model for studying embryonic development (36). While no studies on TULPs in zebrafish have been conducted, several studies have explored the role of p300 in the development of this organism (36, 39). Both genetic knockdown and pharmacological inhibition of p300 in zebrafish embryos result in a severe phenotype characterized by growth retardation, neural malformation, and developmental delay leading to eye, jaw, and heart defects in adults (36). Given the phenotypic similarities between p300 and TULP3 knockdown in multiple organisms, we hypothesized that low levels of TULP3 may underlie some of the effects of p300 inhibition. First, we treated zebrafish embryos with 3 or 5 μM C646 until 24-h post fertilization (hpf). Mirroring our results in mammalian cells, C646 induced a drop in TULP3 protein levels in zebrafish embryos (Fig. 6*A*). This was associated with a marked delay in embryonic development including failure to undergo proper tail extension and head and brain formation in nearly 100% of animals, consistent with previous reports (36) (Fig. 6, *B*–*C*). Next, we designed an intron 3–4 splice-blocking morpholino to knockdown TULP3 and injected it into zebrafish at the single-cell stage. We verified the ability of this morpholino to knockdown TULP3 protein *versus* a control (Fig. 6*D*). In parallel, we coinjected either 100 pg of wild-type TULP3 mRNA or K316Q/K389Q-TULP3 mRNA into knockdown embryos to attempt to rescue any potential phenotypes. We found that TULP3 knockdown mimicked C646 treatment, resulting in developmental delay in a large percentage of the population (Fig. 6, *E*–*F*). In addition, we observed apoptosis in the hindbrain (mild) or hindbrain and forebrain (severe) in roughly 50% of TULP3 knockdown embryos (Fig. 6, *E*–*F*). Importantly, while coinjection with wild-type TULP3 mRNA had relatively little effect on these phenotypes, coinjection with K316Q/K389Q-TULP3 mRNA almost completely reversed the developmental abnormalities (Fig. 6*F*). Indicative of enhanced stability, K316Q/K389Q-TULP3 protein was present at levels approximately fourfold higher than its wild-type counterpart (Fig. 6*G*).

Past work has shown that p300 expression decreases throughout zebrafish development (36). To see if this decrease might be coupled to a decrease in TULP3 protein levels, we performed an immunoblot for p300 and TULP3 protein extracted from untreated zebrafish embryos at several time points. We observed a drop in p300 levels at 24- and 32-h postfertilization in excess of sixfold that was accompanied by a decrease in TULP3 protein (Fig. 6*H*) but not mRNA expression (Fig. S7*B*). Together, these experiments provide evidence that the acetylation switch present on mammalian TULP3 is functionally conserved in zebrafish.

### Protein levels of TULP1, TULP2, and TULP4 are also regulated by acetylation and ubiquitination

**Figure 7:**
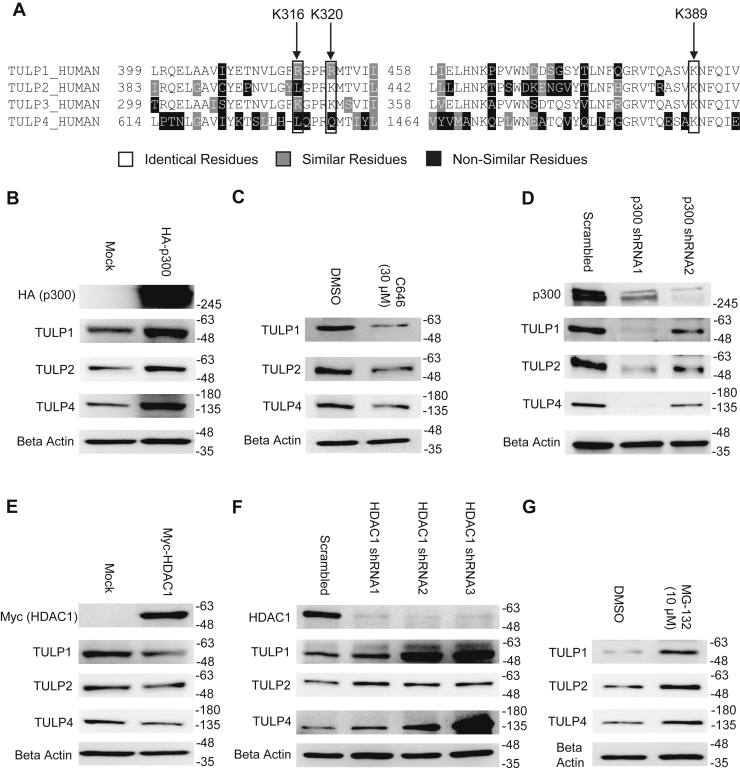
**p300 and HDAC1 influence the stability of multiple tubby-like proteins.***A*, sequence alignment of the four human TULP proteins (TULP1-4) performed using ClustalW software. Identical, similar, and nonsimilar residues are color coded as indicated. Key acetylation sites are indicated with *arrows*. *B*, western blot showing TULP1, TULP2, and TULP4 protein levels in 293T cells transfected with either a control plasmid or a plasmid encoding HA-p300. Cells were harvested 48 h posttransfection. *C*, western blot showing levels of TULP1, TULP2, and TULP4 in 293T following 24 h treatment with either DMSO or 30 μM C646. *D*, western blot of p300, TULP1, TULP2, and TULP4 protein levels following stable transduction of p300 shRNAs into 293T cells. *E*, western blot showing TULP1, TULP2, and TULP4 protein levels in 293T cells transfected with either a control plasmid or a plasmid encoding Myc-HDAC1. Cells were harvested 48 h posttransfection. *F*, immunoblot showing p300, TULP1, TULP2, and TULP4 protein levels following stable transduction of HDAC1 shRNAs into 293T cells. *G*, western blot showing levels of TULP1, TULP2, and TULP4 in 293T cells following 9 h treatment with either DMSO or 10 μM MG-132.

While Lys316 is exclusive to TULP3, Lys320 is partially conserved and Lys389 is fully conserved throughout all tubby-like protein paralogs in mammals (Fig. 7*A*). Given this, we wondered if the acetylation switch on TULP3 might be a conserved mechanism that regulates the stability of one or more additional members of the tubby-like protein family. To examine this assertion, we expressed HA-p300 in 293T cells and measured the levels of TULP1, TULP2, and TULP4 proteins. We found that the protein abundance of all three of these was increased (Fig. 7*B*) to a similar degree as TULP3 (Fig. 2*C*). To extend this analysis further, we treated 293T cells with C646 and performed an immunoblot to measure its effect on TULP1, TULP2, and TULP4 protein levels. Consistent with our previous experiment, we found that all of these proteins were reduced by approximately threefold by treatment with the drug (Fig. 7*C*). A similar decrease in TULP1, TULP2, and TULP4 proteins levels was observed following shRNA knockdown of p300 (Fig. 7*D*). Next, we investigated if HDAC1 might mediate the stability of these TULPs via deacetylation. Expression of Myc-HDAC1 caused a twofold drop in TULP1 and TULP4 protein levels, but had only a marginal effect on TULP2 levels (Fig. 7*E*). Reduction of endogenous basal levels of HDAC1 using lentiviral shRNA caused an increase in TULP1 and TULP4 protein levels but had a modest effect on TULP2, further implying a role for HDAC1 in the regulation of TULP1 and TULP4 but not TULP2 (Fig. 7*F*). Finally, to determine if TULP1, TULP2, and TULP4 were subject to ubiquitination and proteasomal degradation similar to TULP3, we treated cells with MG-132 and measured their protein levels. We observed an approximate twofold increase in the levels of all three proteins in response to the drug, an effect that was slightly more pronounced for TULP1 and TULP4 than TULP2 (Fig. 7*G*). Overall, these results demonstrate that in addition to TULP3, p300 plays a role in modulating protein levels of TULP1, TULP2, and TULP4, while HDAC1 displays differential effects on different TULP paralogs.

## Discussion

Despite their tremendous physiological importance, little work has been done to understand how tubby-like proteins are regulated. Here, we describe a p300/HDAC1-mediated reversible acetylation switch that mediates ubiquitination and subsequent degradation of TULP3 (Fig. S8). There is substantial phenotypic data to support the model that TULP3 acts downstream of p300/HDAC1 and the CRL-3 pathway. First, knockout of p300 in mice is associated with a delay in neural tube closure and ultimately leads to embryonic lethality (40). Moreover, in addition to head, hand, and feet anomalies, polydactyly has been reported in children with Rubinstein–Taybi syndrome (RSTS), a disease caused by mutations in CBP or p300 (41). These phenotypes are astonishingly similar to those displayed by TULP3 knockout mice (21). Second, numerous pieces of evidence point to a critical role for HDAC1 in neural development (42, 43). Third, Culin-3 RING ubiquitin ligase complexes (CRL-3) have been shown to act as master regulators of mammalian cell differentiation and neurogenesis (29). While these effects have classically been attributed to regulation of RhoA protein stability (29), our data detailing an interaction between TULP3 and CUL-3 and its associated Kelch BTB-domain adaptor proteins (KBTB6, KBTB7) (32) (Fig. 1, *B*–*C*), and our data showing an increase in TULP3 protein levels following CUL-3 knockdown, suggest that CRL-3 also regulates TULP3. It is tempting to speculate that lack of TULP3 due to hypoacetylation could underlie many of the developmental defects ascribed to p300 deficiency or aberrant HDAC1 or CRL-3 activity.

While the role of p300 and HDAC1 in controlling gene transcription by regulating the acetylation status of histones, chromatin regulators, and transcription factors is well established (44), less is known regarding how these proteins might quickly transmit signals from the nucleus to the cytoplasm or cilia. Our model (Fig. S8) unveils a new posttranslational signalling pathway that enables rapid cross talk between the nucleus and cilia. In the first step, nuclear TULP3 acts as a sensor for p300/HDAC1 activity and becomes stabilized (acetylated) or marked for degradation (deacetylated). It is interesting to consider that since p300 relies on acetyl-CoA as a cofactor (45), TULP3 acetylation might indirectly be coupled to the metabolic state of the cell. Second, the protein is then shuttled to the cytoplasm where it is either polyubiquitinated by CRL-3 and degraded by the proteasome or binds PIP2 and forms a complex with IFT-A. Finally, the acetylated TULP3/IFT-A complex associates with receptors and mobilizes them to the cilia where they receive extracellular signals. Interestingly, many of the receptors that are trafficked by TULP3 have the ability to transmit signals back to the nucleus (46), allowing for cross talk. For example, activation of GPR161 initiates formation of the Gli3 repressor complex, which shuttles to the nucleus and suppresses transcription of sonic hedgehog target genes (46). This model provides a functional explanation for the presence of TULP3 in the nucleus.

We show that the TULP3 acetylation switch we identify in mammalian cells is also active in zebrafish and that Lys316 and Lys389 are fully conserved among TULP3 orthologs in diverse species (Fig. 6, Fig. S7). While we provide compelling evidence that the residues mediating the acetylation switch are likely confined to the tubby domain (Fig. S6), it is plausible that additional lysine residues, besides those identified in this study, could also play a role in this regulatory mechanism. For example, in addition to the ubiquitination sites mapped in this work, other studies have identified this modification on Lys268 (47, 48), another site that we found to be acetylated (Fig. 2*B*). Future studies could be aimed at examining the role of these additional acetylation and/or ubiquitination sites and determining if the effects of acetylation on ubiquitination are confined to overlapping or adjacent residues on TULP3 or potentially distal sites as well.

The posttranslational regulatory mechanism described in this work applies not only to TULP3, but also to TULP1, TULP2, and TULP4 (Fig. 7). It has been proposed that TULPs, which only exist in eukaryotes, evolved from an ancestral member of the prokaryotic phospholipid scramblase family (PLSCRs) via TUB through divergent evolution and segmental duplication events (4, 49). Owing to conservation of key lysine residues that mediate the acetylation switch (Fig. 7), the effects of p300 acetylation on tubby-like proteins appear to be universal. Thus, this posttranslational pathway has the capacity to regulate the entire protein family in concert. All of the mammalian TULPs, which are expressed in different tissues and under different promoters, play important roles in development (4). Given the complexities of coordinating the transcriptional regulation of these four proteins, we propose that the acetylation switch may have evolved as a mechanism to increase the abundance of all of these proteins together during early embryonic development, in response to rising p300 levels (50). This same mechanism could be used to induce their rapid degradation as p300 levels wane during adulthood (50). This is supported by our data on zebrafish showing that as development proceeds, p300 mRNA and protein levels decrease (36), concomitant with a decrease in TULP3 protein but not mRNA (Fig. 6*H* and Fig. S7*B*). Based on their tissue distributions and substrate specificities, HDACs may superimpose an additional layer of regulation to enable individual targeting of TULPs. While we show that HDAC1 acts as a deacetylase for TULP3, TULP1, and TULP4 (Fig. 4 and Fig. 7), we found that this enzyme did not seem to regulate the stability of TULP2 to an equal extent, suggesting that other deacetylases might be important in different contexts.

Dysregulation of TULPs has been implicated in a number of human diseases including retinitis pigmentosa (TULP1 and TULP2) (4, 51), PKD (TULP3) (11), and several cancers (TULP3) (13, 14). In fact, we queried the COSMIC (the Catalog of Somatic Mutations In Cancer) (52) database for mutations in TULP3 associated with cancer and identified with cancer and identified K320R, a mutation associated with endometrial carcinoma, which corresponds to a site of acetylation that we identified (Fig. 3*A*). Unfortunately, despite their important role in these clinical diseases, TULPs have traditionally been classified as nondruggable due to their lack of enzymatic activity. This study unveils a strategy for controlling TULP protein expression using p300 and HDAC inhibitors that could have therapeutic value (Fig. 2–4, Fig. 7). For example, HDAC1 inhibitors could be used to boost TULP3 levels in diseases caused by hypomorphic alleles such as K407I-TULP3, which is associated with PKD (11). The fact that the safety of HDAC inhibitors in humans has already been established in clinical trials for several cancers (53) could facilitate their repurposing for TULP-related disorders. Alternatively, small-molecule p300 activators, such as CTPB (N-(4-chloro-3-trifluoromethyl-phenyl)-2-ethoxy-6-pentadecyl-benzamide) (54), could be used to perform the same function while p300 inhibitors could be used to treat diseases caused by TULP gain of function. Future studies could be designed to test the viability of this approach.

This study sheds light on the posttranslational regulation of TULP3 and its related tubby-like proteins by identifying a conserved p300/HDAC1-mediated acetylation switch that modulates the protein levels of this family. In addition, it describes a pathway that enables cross talk between chromatin factors in the nucleus and a wide range of receptors localized to cilia. Finally, it details a new pharmacological strategy for globally controlling TULP protein stability. We anticipate that these findings will be of ongoing significance as the list of physiological functions and disease-causing mutations attributed to tubby-like proteins continues to expand.

## Experimental procedures

### Experimental models

This project used two female human cell lines, HEK293T cells and HeLa cells that were originally obtained from the American Type Culture Collection (ATCC) and were tested and found to be *mycoplasma*-free. Cells were cultured in a humidified atmosphere with 5% CO_2_ at 37 °C. In addition, this project used unisex zebrafish (sex undifferentiated). All animal procedures used protocols that were approved by the University of Alberta Animal Care and Use Committee, Biosciences (#0082).

### Reagents

Rabbit anti-TULP1 (ab97281) and rabbit anti-TULP3 antibody (ab155317) were obtained from Abcam. HDAC Inhibitor XXIV (OSU-HDAC-44) was obtained from Calbiochem. Cycloheximide (2112S), MG-132 (2194S), rabbit antiacetylated lysine (#9441S), rabbit anti-beta actin (13E5, #4970S), rabbit anti-CUL3 (#2759S), rabbit anti-HA tag (C29F4, #3724T), mouse anti-HDAC1 (10E2, #5356S), antimouse IgG, HRP linked antibody (7076S), rabbit anti-Myc tag (71D10, #2278S), rabbit anti-P300 (D1M7C, #70088S), rabbit anti-Rad18 (D2B8, #9040S), rabbit anti-SIRT1 (C14H4, #2496S), anti-rabbit IgG, HRP linked antibody (7074S), rabbit antiubiquitin (#3933S), Trichostatin A (9950S) were obtained from Cell Signaling Technology, Inc. Rabbit anti-PP6R3 (16944-1-AP) was obtained from Proteintech. Anacardic Acid (A7236), Anti-FLAG (M2, #F1804), C646 (SML0002), EX-527 (E7034), MC1568 (M1824), mouse antiacetyl lysine (4G12, 05-515), mouse anti-TULP2 (2B5, WH0007288M3), MS-275 (EPS002), and nicotinamide (N3376) were obtained from Sigma Aldrich.

### Plasmids, cloning, and site-directed mutagenesis

HDAC1-FLAG was a gift from Eric Verdin (Addgene plasmid # 13820; http://n2t.net/addgene:13820; RRID: Addgene_13820). pCMVβ-p300-myc was a gift from Tso-Pang Yao (Addgene plasmid # 30489; http://n2t.net/addgene:30489; RRID: Addgene_30489). pMD2.G was a gift from Didier Trono (Addgene plasmid # 12259; http://n2t.net/addgene:12259; RRID: Addgene_12259). psPAX2 was a gift from Didier Trono (Addgene plasmid # 12260; http://n2t.net/addgene:12260; RRID: Addgene_12260). N-terminally tagged FLAG- TULP3 was cloned into the retrovirus compatible MSCV vector using the Xho1 restriction site. Successful clones were screened by Sanger sequencing. Site-directed mutagenesis was performed using the Q5 site-directed mutagenesis kit according to the manufacturer′s instructions.

### Cell culture and transfection

HEK293T and HeLa cells were maintained in DMEM (11995-065, Gibco) supplemented with 10% fetal bovine serum (FBS) (F1051, Sigma Aldrich), 1% Penicillin Streptomycin (15140-122, Gibco), and 1% Glutamine (25030-081, Gibco). Cells were cultured in a 37 °C/5% CO2 incubator with 97% humidity. For transfection, cells were seeded the day before transfection at 25% confluency using a Z2 Coulter Particle Count and Size Analyzer (Beckman Coulter). HEK293T cells were transfected with Effectene Transfection Reagent (Qiagen) according to the manufacturer′s instructions. HeLa cells were transfected with Lipofectamine 3000 (Invitrogen) according to the manufacturer′s instructions. siRNAs were transfected using Lipofectamine RNAiMAX according to the manufacturer’s instructions. Cells were harvested 48-h posttransfection (72 h for siRNA experiments).

### Identification of TULP3 protein interactors by IP-LC-MS/MS

Lysates from HEK293T cells stably expressing either empty MSCV or MSCV FLAG-TULP3 were harvested, normalized for protein concentration, and subject to immunoprecipitation using anti-FLAG beads as described above. Subsequently, SDS-PAGE was performed using equal volumes of mock-FLAG and FLAG-TULP3 samples followed by either silver staining using the Pierce Silver Stain Kit (24612, ThermoFisher) (for gel analysis) or Coomassie staining using BioSafe Coomassie G-250 (for mass spectrometry identification). Protein bands of interest were excised from the Coomassie-stained gel and in-gel trypsin digestion on the samples was performed. The tryptic peptides were resolved and ionized by using nanoflow-HPLC (Thermo Scientific Easy-nLC 1000 system) coupled to a Q Exactive Orbitrap mass spectrometer (Thermo Scientific) with C18 columns described above. Data was processed using Proteome Discoverer 1.4 (Thermo Scientific), and the uniport human database (2016–11–26) was searched using SEQUEST (Thermo Scientific). Search parameters included a strict FDR of 0.01 using a decoy database, a relaxed FDR of 0.05, a maximum of three missed trypsin cleavages, a precursor mass tolerance of 10 ppm and a fragment mass tolerance of 0.01 Da, constant modification carbamidomethylation (C), and variable modifications of deamidation (N/Q) and oxidation (M). The maximum number of variable modifications was set to 4.

### Coimmunoprecipitation experiments

Following transfection with the appropriate plasmids, cells were harvested and washed with ice cold PBS and lysed on a rotator for 30 min at 4 °C with protein lysis buffer: 1% Triton X-100, 50 mM Tris-HCl pH 8.0, 150 mM NaCl supplemented with complete ultra protease inhibitor cocktail (05892791001, Roche). Cell lysates were centrifuged at 13,000 rpm for 15 min, and the supernatants were added to EZview Red ANTI-FLAG M2 Affinity Gel or EZview Red ANTI-HA Affinity Gel and immunoprecipitated on a rotator for 2 h at 4 °C. The beads were then washed five times with protein lysis buffer and eluted with 100 μg/ml 3x FLAG peptide on a rotator for 1 h. Laemmli buffer was added to samples prior to gel electrophoresis and western blotting.

### Acetylation and ubiquitination experiments in cells

HEK293T cells stably expressing FLAG-TULP3 were transfected with the indicated plasmids for 24 or 48 h. For ubiquitin experiments, 10 μM MG-132 was added to cells 9 h before harvesting to allow ubiquitinated TULP3 to accumulate. Subsequently, cells were washed with PBS and lysed in buffer containing 8 M urea, 150 mM NaCl, and 50 mM Tris-HCl pH 8.0 and 1% NP40. Genomic DNA was sheared by passing the lysate through a 22-gauge needle 20 times. Cell lysates were then diluted tenfold in protein lysis buffer and immunoprecipitated as described. For acetylation experiments, cells were washed in PBS and lysed in protein lysis buffer supplemented with 20 mM Nicotinamide (NAM) and 1 μM Trichostatin A (TSA), used during subsequent washes as well. Samples were then analyzed by gel electrophoresis and western blotting. Five percent BSA in TBST was used for all stages of ubiquitin and acetylation immunoblotting. For immunoprecipitation experiments, protein equalization was performed prior to loading on a gel to ensure that an equal amount of TULP3 protein was present across all experimental groups, facilitating comparison.

### Analysis of acetyl-lysine modifications by LC-MS/MS

HEK293T cells stably expressing FLAG-TULP3 were transfected with plasmids encoding tagged p300, PCAF, or GCN5 acetyltransferases and harvested as described above but with protein lysis buffer supplemented with 20 mM NAM and 1 μM TSA to inhibit deacetylases. FLAG-TULP3 was then immunoprecipitated as described above and subject to SDS-PAGE followed by staining with Coomassie G-250 according to the manufacturer′s instructions. In short, peptides were separated using a nanoflow-HPLC coupled to a LTQ Orbitrap Mass Spectrometer (Thermo Fisher Scientific). Data were analyzed using Sequest (Sequest ver 28 rev 13) against the TULP3 sequence (uniprot/O75386). Search parameters included a precursor mass tolerance of 50 ppm, a fragment mass tolerance of 1 Da, with the constant modification carbamidomethylation (C), and variable modifications of oxidation (M), phosphorylation (STY), and/or acetyl (uncleaved K). The maximum number of variable modifications was set to 6. The results can be found in Supplemental Files 2 and 3.

### Real-time qRT-PCR

RNA from HEK293T cells or zebrafish embryos was extracted using TRIzol Reagent (Invitrogen) according to the manufacturer’s instructions and quantified using a Nanophotometer NP80 (Implen). For mammalian cell work, qRT-PCR reactions were performed using the iTaq Universal SYBR Green One-Step Kit (BioRad) while zebrafish reactions were carried out using the iScript cDNA Synthesis Kit (BioRad) and iQ SYBR Green Supermix (BioRad). Reactions were run on a CFX96 C1000 Touch real-time instrument (BioRad). Calculations were performed using a comparative method (2^−ΔΔCT^) using Beta Actin mRNA and 18S rRNA loading controls for HEK293T cells and zebrafish lysates, respectively.

### Viral production, transduction, and siRNA experiments

For experiments employing shRNAs (Dharmacon Horizon Discovery), human pLKO.1 lentiviral vectors were cotransfected with pMD2.G and psPAX2 plasmids into HEK293T cells. The following shRNA sequences were used: HDAC1 shRNA 1 (TRCN0000004814) 5′-TATGGTTCAAAGTTAAGAACG-3′, HDAC1 shRNA 2 (TRCN0000004815) 5′-TTACGAATGGTGTAACCACCG-3′, HDAC1 shRNA 3 (TRCN0000004816) 5′-ATTACTTTGGACATGACCGGC-3′, HDAC1 shRNA 4 (TRCN0000004817) 5′-AAGTTGGAAGAGTTCTTGCGG-3′, EP300 shRNA 1 (TRCN0000009882) 5′-TACCATGCCAAGACTTGTCTG-3′, EP300 shRNA 2 (TRCN0000009883) 5′-TCTCAAGATGTCTCGGAATTG-3′, EP300 shRNA 3 (TRCN0000039885) 5′-ATGTCTCGGAATTGTGAAGGC-3′, TRC Non-targeting control shRNA (RHS6848) 5′- CCGGTTGGTTTACATGTTGTGTGACTCGAGTCACACAACATGTAAACCATTTTTG-3′

To generate FLAG-TULP3 retroviral particles, the Murine Stem Cell Virus (MSCV) vector was cotransfected alongside VSV-G and GAG-Pol plasmids into HEK293T cells. For both sets of experiments, viral supernatants were collected 48 h posttransfection and supplemented with 10 μg/ml polybrene prior to being added to HEK293T or HeLa cells for 24 h. Transduced cells were selected by incubating cells in the presence of 2 μg/ml puromycin for three days. Stable transduction was verified by western blot. siRNA for Cul-3 knockdown was ordered as a SMARTPool (Dharmacon Horizon Discovery M-010224-02-0005) and transfected using Lipofectamine RNAiMAX into 293T cells. Protein levels were analyzed 72 h posttransfection by western blot.

### Cycloheximide pulse-chase experiments

HEK293T cells stably transduced with FLAG-TULP3 or the respective mutant proteins were seeded at 66% confluency the day before the cycloheximide (CHX) chase. In total, 150 μg/ml CHX dissolved in DMSO was added to cells, which were then harvested and lysed at different timepoints, as described.

### Nuclear/cytoplasmic cell fractionation

The day before fractionation, HEK293T cells were seeded at 75% confluency using a Z2 Coulter Particle Count and Size Analyzer (Beckman Coulter) on a 60 mm plate. Cells were then harvested and washed twice in ice-cold PBS with Ca and Mg. The crude cytoplasmic fraction was prepared by adding 400 uL of PBS with Ca/Mg, 0.27 M sucrose, 6.25% Ficoll PM 400 (F4375, Sigma Aldrich), and 0.1% NP40 and gently resuspending the cell pellet followed by 5 min of centrifugation at 1300*g* at 4 °C. The supernatant containing cytoplasmic proteins was collected into a fresh microcentrifuge tube while the nuclear pellet was washed in an additional 500 uL of lysis buffer and centrifuged at 1300*g* for 5 min. The crude cytoplasmic fraction was then centrifuged at 13,000 rpm for 10 min to remove residual nuclei, and the supernatant was collected as the final cytoplasmic fraction. The washed nuclear pellet was then lysed in 100 μl RIPA buffer by vortexing at maximum speed every 10 min for 1 h on ice. Nuclear debris was pelleted by centrifuging at 13,000 rpm for 10 min after which the supernatant was collected and moved to a fresh microcentrifuge tube as the final nuclear fraction. Cytoplasmic and nuclear samples were quantified using the Pierce BCA Protein Assay kit and equalized prior to being subject to gel electrophoresis and western blotting. Lamin A/C and alpha tubulin were used as standards to assess nuclear and cytoplasmic fraction purity, respectively.

### Analysis of ubiquitin modifications by LC-MS/MS

HEK293T cells stably expressing FLAG-TULP3 were treated with 10 μM MG-132 for 9 h to allow ubiquitylated TULP3 to accumulate prior to lysis and immunoprecipitation. FLAG-TULP3 protein was eluted from FLAG-beads by boiling in Laemmli buffer, run on an SDS-PAGE gel, and subjected to a Blue-Silver stain, according to the manufacturer’s instructions. Each gel lane was cut into four pieces and destained in a solution of 50 mM ammonium bicarbonate/50% acetonitrile at 37 °C. Subsequently, gel pieces were dried by incubating with acetonitrile at 37 °C, rehydrated and reduced with 175 μl of reducing solution (5 mM β-mercaptoethanol, 100 mM ammonium bicarbonate) at 37 °C for 30 min, and alkylated with 175 μl of alkylating solution (50 mM iodoacetamide, 100 mM ammonium bicarbonate) at 37 °C for 30 min. The gel pieces were washed twice with 175 μl of 100 mM ammonium bicarbonate at 37 °C for 10 min and completely dried by incubating with acetonitrile at 37 °C. Proteins in each well were digested using 1 μg of sequencing-grade trypsin (Promega Inc.) in 75 μl of 50 mM ammonium bicarbonate and incubated overnight. Tryptic peptides in the gel pieces were extracted by incubating with 2% acetonitrile, 1% formic acid, then with 50% acetonitrile, 0.5% formic acid, each at 37 °C for 1 h. The extracted peptides were transferred to another round-bottom 96-well plate, dried using a Genevac (EZ-2 plus). For *in vitro* assays, fractionated peptides in the same sample were combined to be injected onto MS together. Peptides were separated using a nanoflow-HPLC (Thermo Scientific EASY-nLC 1200 System) coupled to Orbitrap Fusion Lumos Tribrid Mass Spectrometer (Thermo Scientific). A trap column (5 μm, 100 Å, 100 μm × 2 cm, Acclaim PepMap 100 nanoViper C18; Thermo Scientific) and an analytical column (2 μm, 100 Å, 50 μm × 15 cm, PepMap RSLC C18; Thermo Scientific) were used for the reverse-phase separation of the peptide mixture. Peptides were eluted over a linear gradient over the course of 90 min from 3.85% to 36.8% acetonitrile in 0.1% formic acid. The peaklists were generated with ProteoWizard (msconvert, v3.0.20044-d751fcb4e) (55) and analyzed using ProteinProspector (v5.22.1) against the TULP3 sequence (uniprot/O75386). Search parameters included a maximum of three missed trypsin cleavages, a precursor mass tolerance of 15 ppm, a fragment mass tolerance of 0.8 Da, with the constant modification carbamidomethylation (C), and variable modifications of acetylation (protein N-term), deamidation (N/Q), oxidation (M), GlyGly (uncleaved K), or acetylation (uncleaved K). The maximum number of variable modifications was set to 4. The results can be found in Supplemental Files 2 and 3.

### *In vitro* acetylation assay

In total, 0.5 μg of recombinant human TULP3 protein (H00007289-P01, Novus Biologicals) was incubated in the absence or presence of 0.5 μg of recombinant human p300 (catalytic domain) (BML-SE451-0100, Enzo Life Sciences), 20 μM acetyl-coA, or 30 μM C646, as indicated, for 1 h at 37 °C. Samples were then prepared for in-gel trypsin digestion and mass spectrometry as described above.

### Zebrafish husbandry and animal care

All experiments conducted were approved by the University of Alberta Animal Care and Use Committee, Biosciences (#0082). Anesthesia and euthanasia were performed with tricaine methanesulfonate (Syndel). Adult and embryonic zebrafish were cared for according to standard protocols. Embryos were grown at 28.5 °C in E3 embryo media. Developing embryos were staged according to standard morphological milestones (56). The AB strain was used as the wild-type strain.

### Pharmacological treatment and imaging of zebrafish embryos

Zebrafish embryos were raised to 70% epiboly, dechorionated with pronase E (Sigma-Aldrich), and treated until 24 hpf with the indicated concentration of C646 (Sigma-Aldrich) dissolved in E3 media. Phenotypic analysis was performed on anesthetized embryos at 24 hpf using an Olympus stereomicroscope (SZX-12).

### Morpholino design

A sequenced transcript (zmp:0000000711; ENSDART00000093236.6) previously identified to be orthologous to human TULP3 was used to design a morpholino to target zebrafish *tulp3*. A splice-blocking morpholino (5ʹ-GCCCTCTGTCAATGCACAAACACTG-3ʹ; Gene Tools LLC) was designed to the boundary of the 3ʹ splice acceptor of intron 3 and exon 4. Eight nanograms of *tulp3* morpholino was coinjected with 2 ng of zebrafish p53 morpholino (5ʹ-GCGCCATTGCTTTGCAAGAATTG-3ʹ) at the one-cell stage. A standard control morpholino (5ʹ-CCTCTTACCTCAGTTACAATTTATA-3ʹ; Gene Tools LLC) was used as a negative control.

### Generation of zebrafish overexpressing TULP3 mRNA and microinjection

Wild-type and K316Q/K389Q variant human TULP3 sequences were cloned and ligated into pCS2+ vectors. Following linearization with NotI (NEB), mRNA was transcribed using the mMachine SP6 Transcription Kit (Invitrogen) and purified using Amicon Ultra-0.5 Centrifugal Filter columns (Ultracel-50; Fisher/Millipore). Wild-type zebrafish embryos were injected with either 100 pg of wild-type TULP3 or the K316Q/K389Q mutant TULP3 mRNA at the one-cell stage. *gfp* mRNA was prepared as above and used as a negative control.

### Quantification and statistical analysis

Numbers of trial replicates and appropriate statistical measures and tests are denoted in Figure captions.

## Data availability

All data relating to this study are included in the article and supporting information files. Inquiries relating to this study and reagent requests should be directed to the corresponding author, Dr Basil P. Hubbard (bphubbard@ualberta.ca). All new plasmid constructs generated from this project will be made publicly available on addgene (www.addgene.org). All LC-MS/MS raw files have been deposited in the MassIVE repository (massive.ucsd.edu) and are freely available: MSV000086358 (Fig. 2*B*), MSV000086346 (Fig. 5*E*), MSV000086357 (Supplemental File 1) and MSV000086340 (Fig. S1*F*).

## Conflict of interest

The authors declare that they have no conflicts of interest with the contents of this article.
