# Supplementary material for: A conserved acetylation switch enables pharmacological control of tubby-like protein stability
Source: J Biol Chem. 2020 Nov 23;296:100073. doi: 10.1074/jbc.RA120.015839 (PMC7948452; doi:10.1074/jbc.RA120.015839)
Supplement: Figures S1–S7 [file mmc1.pdf]

A conserved acetylation switch enables pharmacological control of tubby-like protein stability

Evan M. Kerek, Kevin H. Yoon, Shu Y. Luo, Jerry Chen, Robert Valencia, Olivier Julien, Andrew J. Waskiewicz, Basil P. Hubbard

Supporting Information

**Supplemental Figure 1**	TULP3 interacts with SIRT1 and p300, which alters its protein levels and acetylates it.
**Supplemental Figure 2**	Intracellular localization of wild-type and acetylation-mutant TULP3 proteins.
**Supplemental Figure 3**	Stability and baseline expression of TULP3 acetylation-mutant proteins.
**Supplemental Figure 4**	Inhibition of sirtuin activity does not alter TULP3 protein levels.
**Supplemental Figure 5**	TULP3 is polyubiquitinated in cells and Lys316 and Ly389 are important for this modification.
**Supplemental Figure 6**	Deletion of the tubby domain renders TULP3 refractory to the effects of C646 and MG-132.
**Supplemental Figure 7**	A TULP3 acetylation switch is conserved in zebrafish.
**Supplemental Figure 8**	Model illustrating a TULP3 acetylation/ubiquitination pathway that spans both cytoplasmic and nuclear compartments.

Supplemental Figure 1

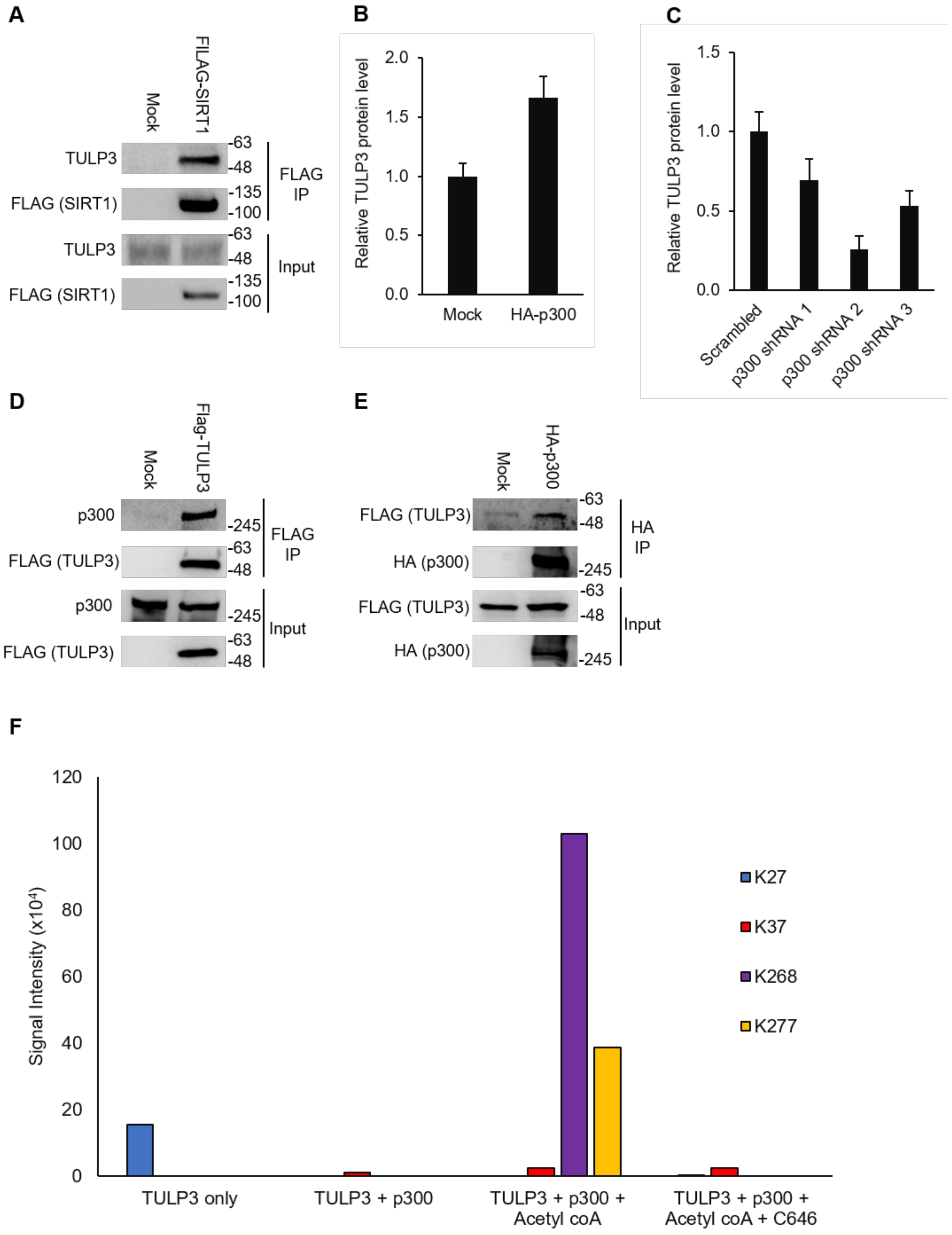

Figure S1. TULP3 interacts with SIRT1 and p300, which alters its protein levels and acetylates it. **(A)** Western blots showing co-immunoprecipitation of FLAG-SIRT1 with TULP3. Densitometry quantification of endogenous levels of TULP3 in HeLa cells corresponding to **(B)** samples in **Fig. 2C** and **(C) Fig. 2E** performed using ImageJ; n=3 biological replicates, Mean ± S.D. shown. **(D)** Immunoblots showing co-immunoprecipitation of FLAG-TULP3 with p300 in 293T cells. **(E)** Immunoblots showing co-immunoprecipitation of HA-p300 with FLAG-TULP3 in 293T cells. **(F)** Bar graph illustrating the presence of acetylation on recombinant TULP3, as detected by semi-quantitative LC-MS/MS, following in vitro acetylation reactions using the indicated conditions. The concentrations of acetyl-CoA and C646 used in the reactions were 20 μM and 30 μM, respectively.

Supplemental Figure 2

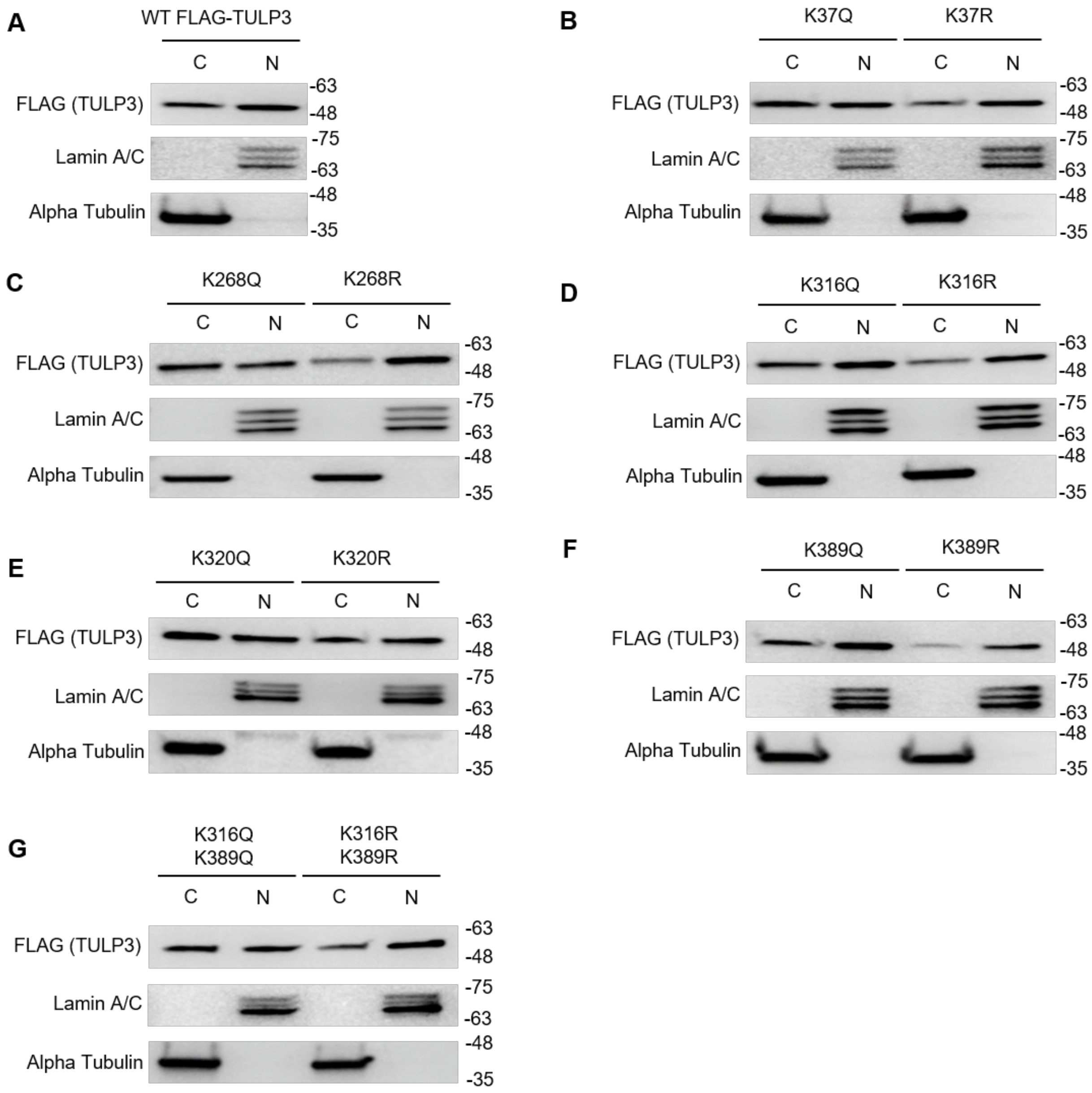

Figure S2. Western blots showing the cytoplasmic and nuclear fractions of **(A)** wild-type FLAG-TULP3, or **(B)** K37Q/K37R, **(C)** K268Q/K268R, **(D)** K316Q/K316R, **(E)** K320Q/K320R, **(F)** K389Q/K389R, **(G)** K316Q/K389Q and K316R/K389R mutant proteins in HEK293T cells. Lamin A/C and alpha tubulin were used as nuclear and cytoplasmic markers, respectively.

Supplemental Figure 3

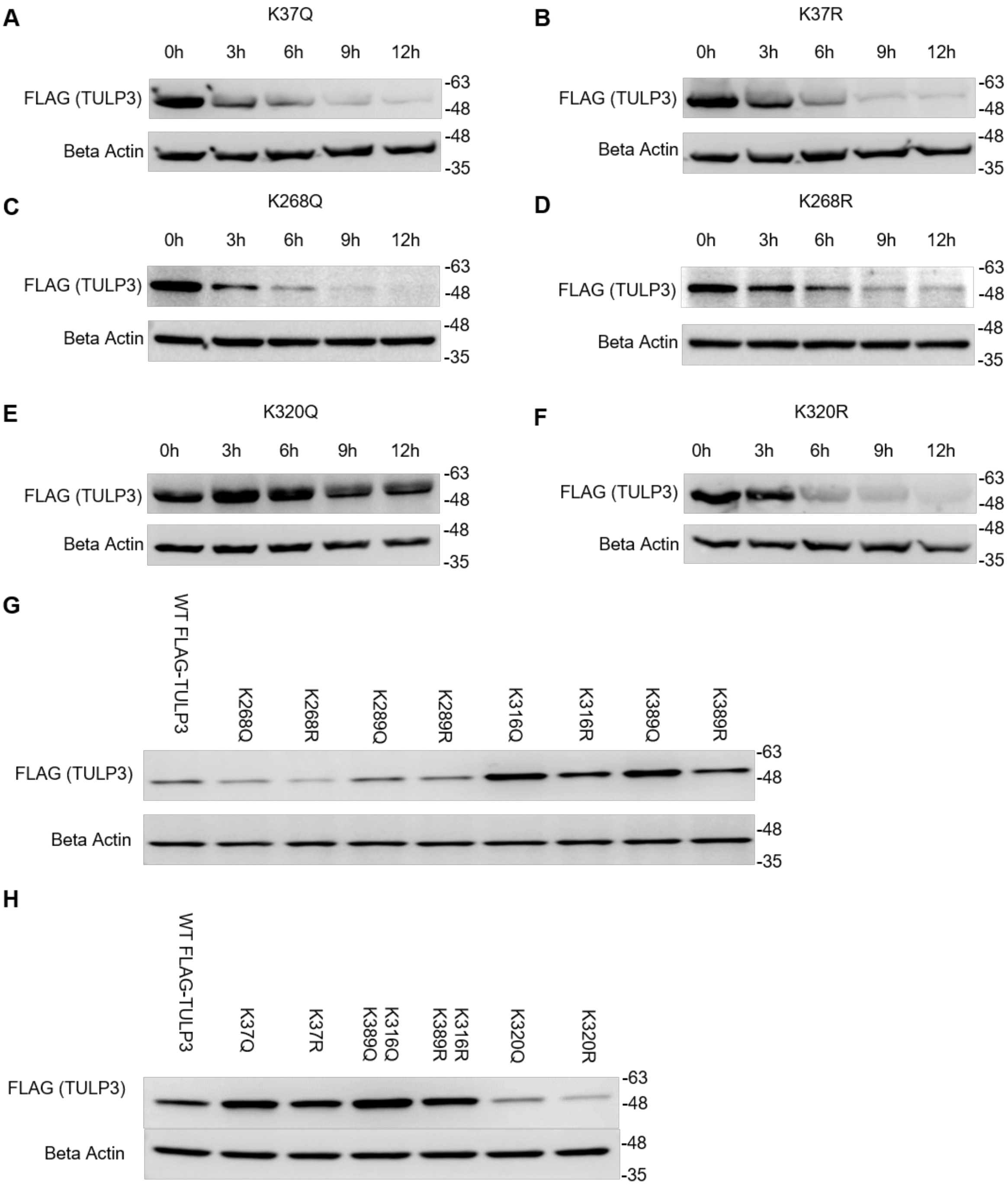

Figure S3. Stability and baseline expression of TULP3 acetylation-mutant proteins. Western blots showing cycloheximide pulse-chase experiments of **(A)** FLAG-TULP3 (K37Q), **(B)** K37R, **(C)** K268Q**,** **(D)** K268R, **(E)** K320Q, and **(F)** K320R stably expressed in 293T cells. Cells were harvested at the indicated timepoints. **(G, H)** Baseline expression of stably expressed wild-type FLAG-TULP3 or mutant proteins in 293T cells.

Supplemental Figure 4

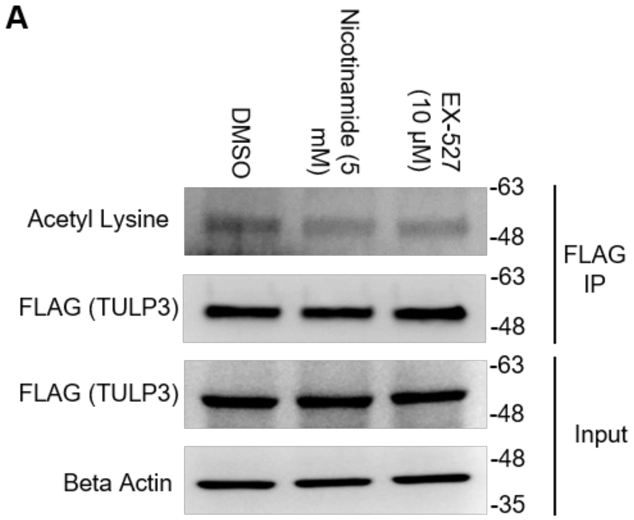

Figure S4. Inhibition of sirtuin activity does not alter TULP3 protein levels. (A) Western blot showing total FLAG-TULP3 protein and acetylation levels in 293T cells following 24 hours of treatment with DMSO, 5 mM NAM, or 10 μM EX-527.

Supplemental Figure 5

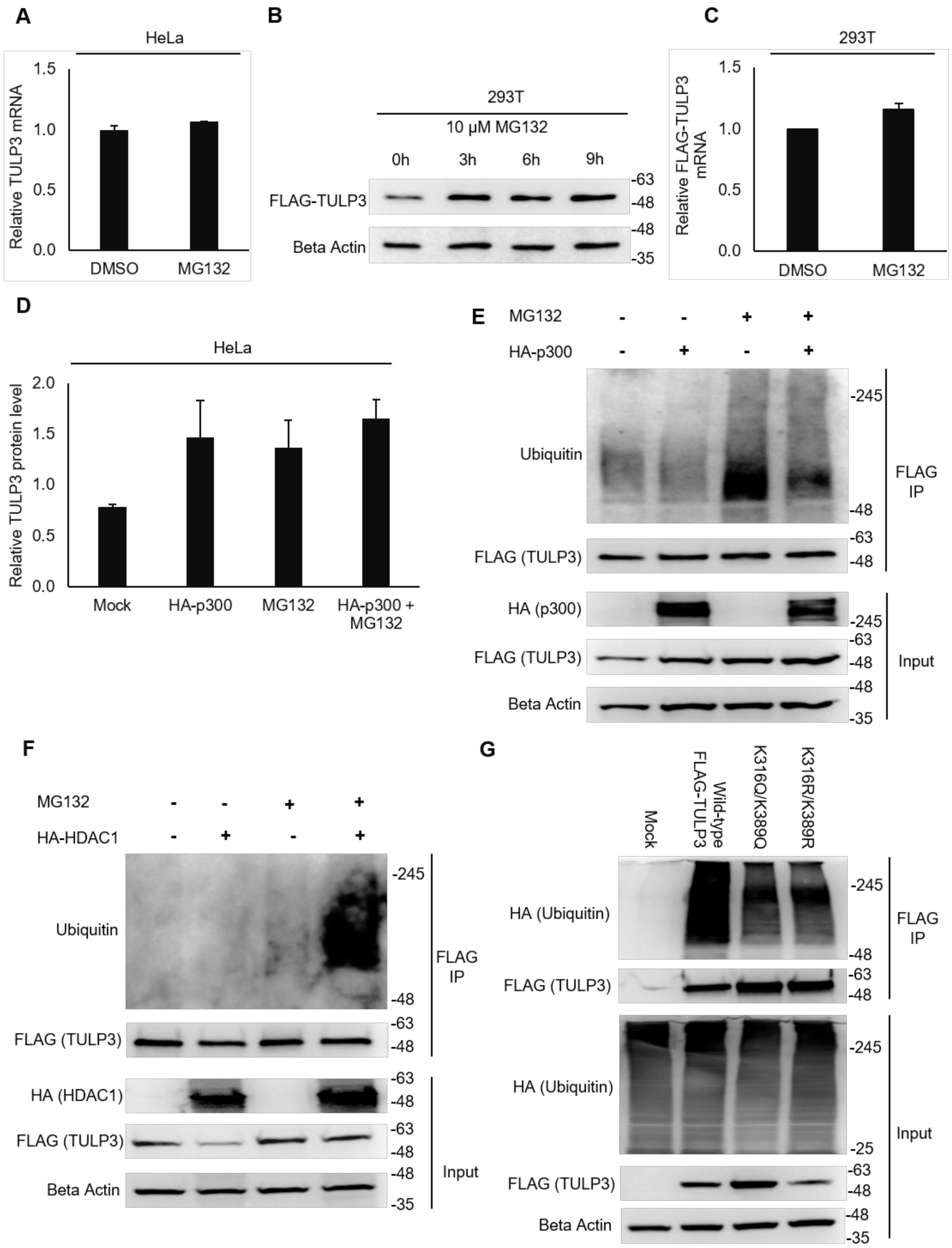

Figure S5. TULP3 is polyubiquitinated in cells and Lys316 and Ly389 are important for this modification. (A) mRNA levels of endogenous TULP3 in HeLa cells corresponding to samples in **Fig. 5A** examined using qRT-PCR; n=4 technical replicates, Mean ± S.D. shown. **(B)** Western blot showing FLAG-TULP3 protein levels in 293T cells following treatment with 10 µM MG-132 for the indicated intervals. **(C)** mRNA levels of FLAG-TULP3 in 293T cells corresponding to samples in **(B)** examined using qRT-PCR; n=4 technical replicates, Mean ± S.D. shown. **(D)** Densitometry quantification of endogenous protein levels of TULP3 in HeLa cells corresponding to samples in **Fig. 5B** performed using ImageJ; n=3 biological replicates, Mean ± S.D. shown. **(E)** Immunoblot analysis of ubiquitination levels on FLAG-TULP3 immunoprecipitated from 293T cells in the absence or presence of 10 µM MG-132 and transfected with a control plasmid or a plasmid encoding HA-p300 as indicated. Cells were harvested 48h post-transfection following 9h of MG-132 treatment. **(F)** Western blot analysis of ubiquitination levels on FLAG-TULP3 immunoprecipitated from 293T cells in the absence or presence of 10 µM MG-132 and transfected with a control plasmid or a plasmid encoding HA-HDAC1 as indicated. Cells were harvested 48h post-transfection following 9h of MG-132 treatment. **(G)** Western blot showing total protein levels and ubiquitination status of stably expressed Flag-TULP3, Flag-TULP3 K316Q/K389Q, and Flag-TULP3 K316R/K389R in 293T cells transfected with HA-ubiquitin following 9h treatment with 10 µM MG-132. Cells were harvested 48h post-transfection.

Supplemental Figure 6

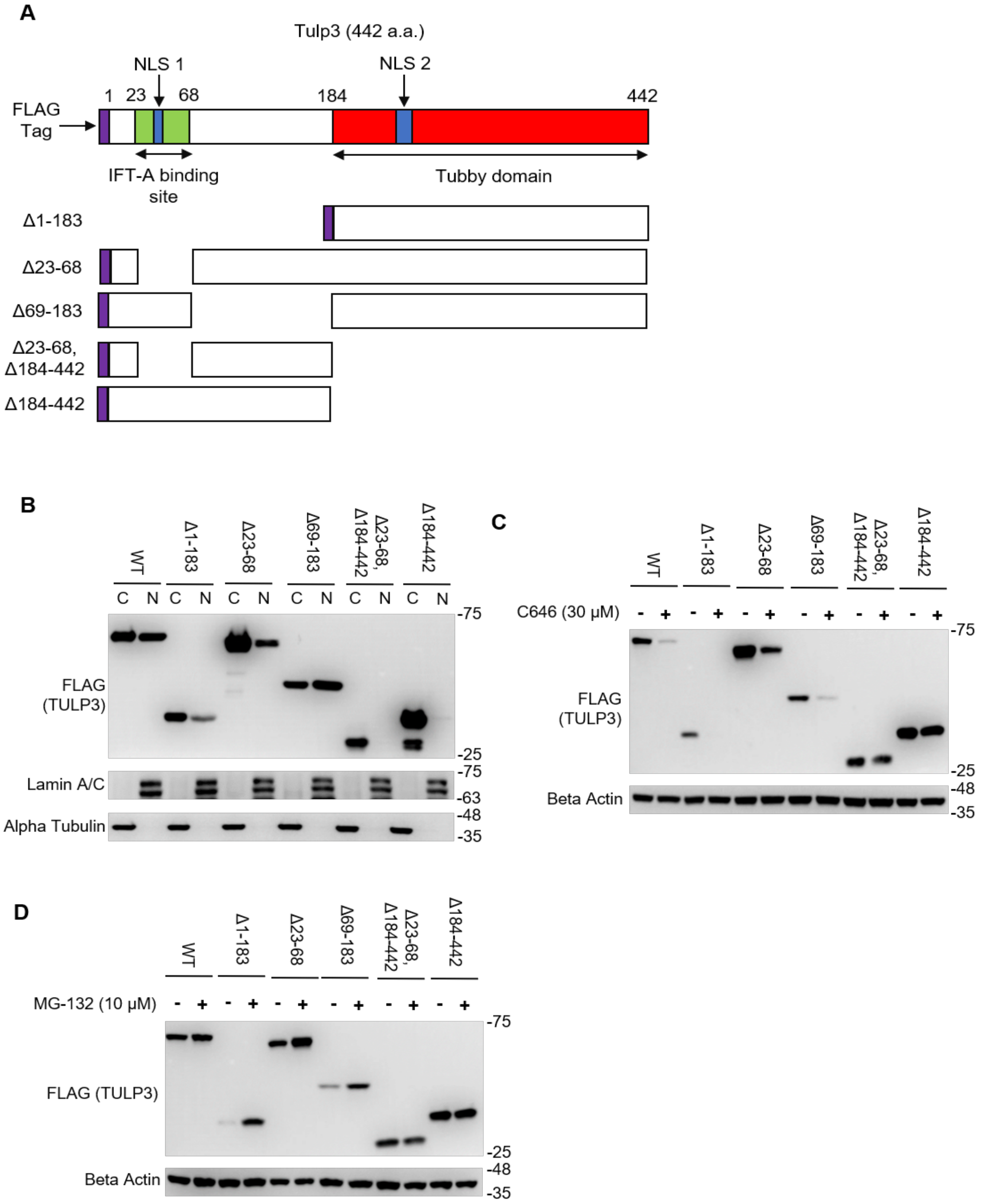

Figure S6. Deletion of the tubby domain renders TULP3 refractory to the effects of C646 and MG-132. (A) Schematic depicting functional motifs and domains in TULP3. Amino acids corresponding to deleted regions in variant constructs have been numbered. IFT-A ID denotes Intraflagellar Transport Complex A interacting domain, while NLS denotes Nuclear Localization Sequence (predicted using cNLS Mapper). **(B)** Immunoblot outlining the sub-cellular localization of wild-type FLAG-TULP3 and several truncation and deletion variants in 293T cells. Alpha tubulin and Lamin A/C were used as cytoplasmic and nuclear marker proteins, respectively. **(C)** Western blot showing the total protein levels of FLAG-TULP3 or several truncation or deletion variants in 293T cells treated with DMSO or 30 µM C646 for 24h. **(D)** Western blot showing the total protein levels of FLAG-TULP3 or several truncation or deletion variants in 293T cells treated with DMSO or 10 µM MG-132 for 9h.

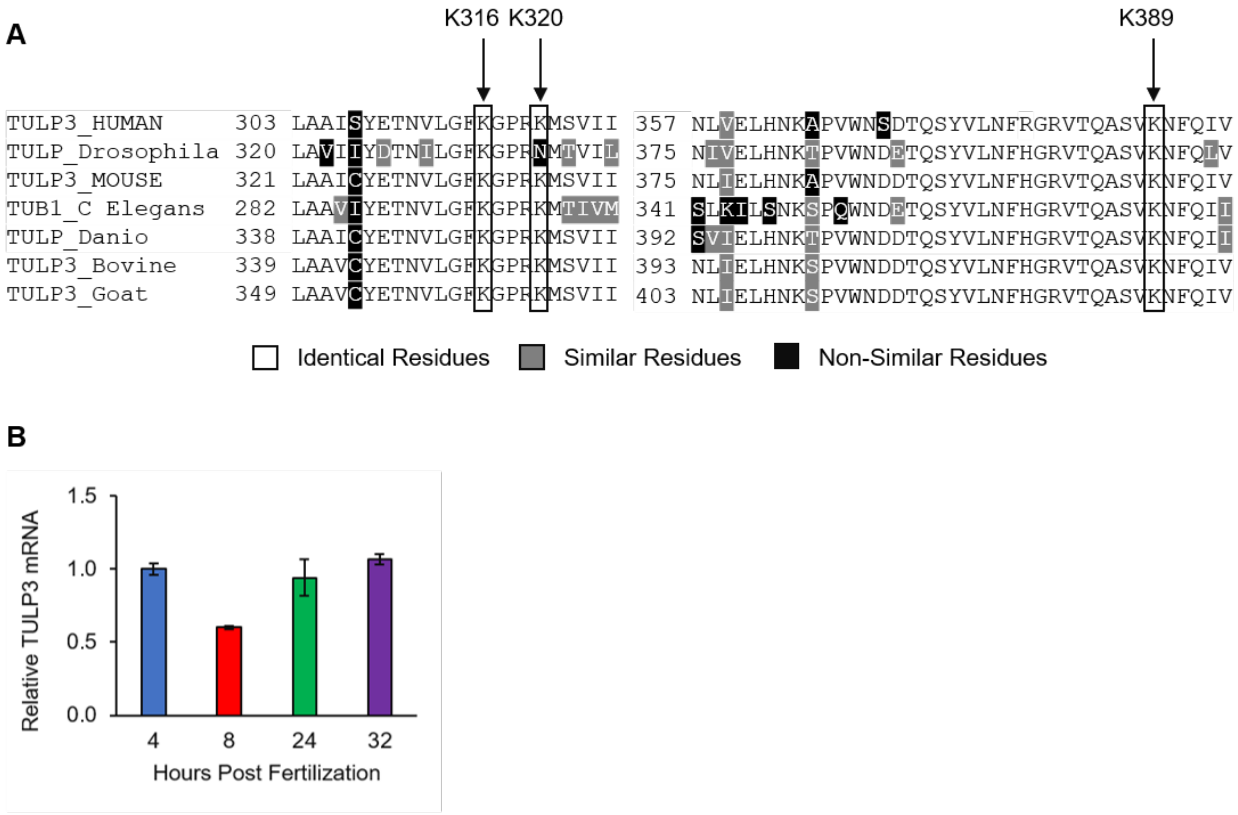

Figure S7. A TULP3 acetylation switch is conserved in zebrafish. (A) Sequence alignment of TULP3 orthologs in diverse species performed using ClustalW software. Identical, similar, and non-similar residues are color coded as indicated. Key acetylation sites are indicated with arrows. **(B)** mRNA levels of TULP3 analyzed using quantitative real-time PCR from zebrafish embryos harvested at 4, 8, 24- and 32-hours post fertilization. Results are normalized to 18S RNA; n=3 biological replicates; Mean ± SEM shown.

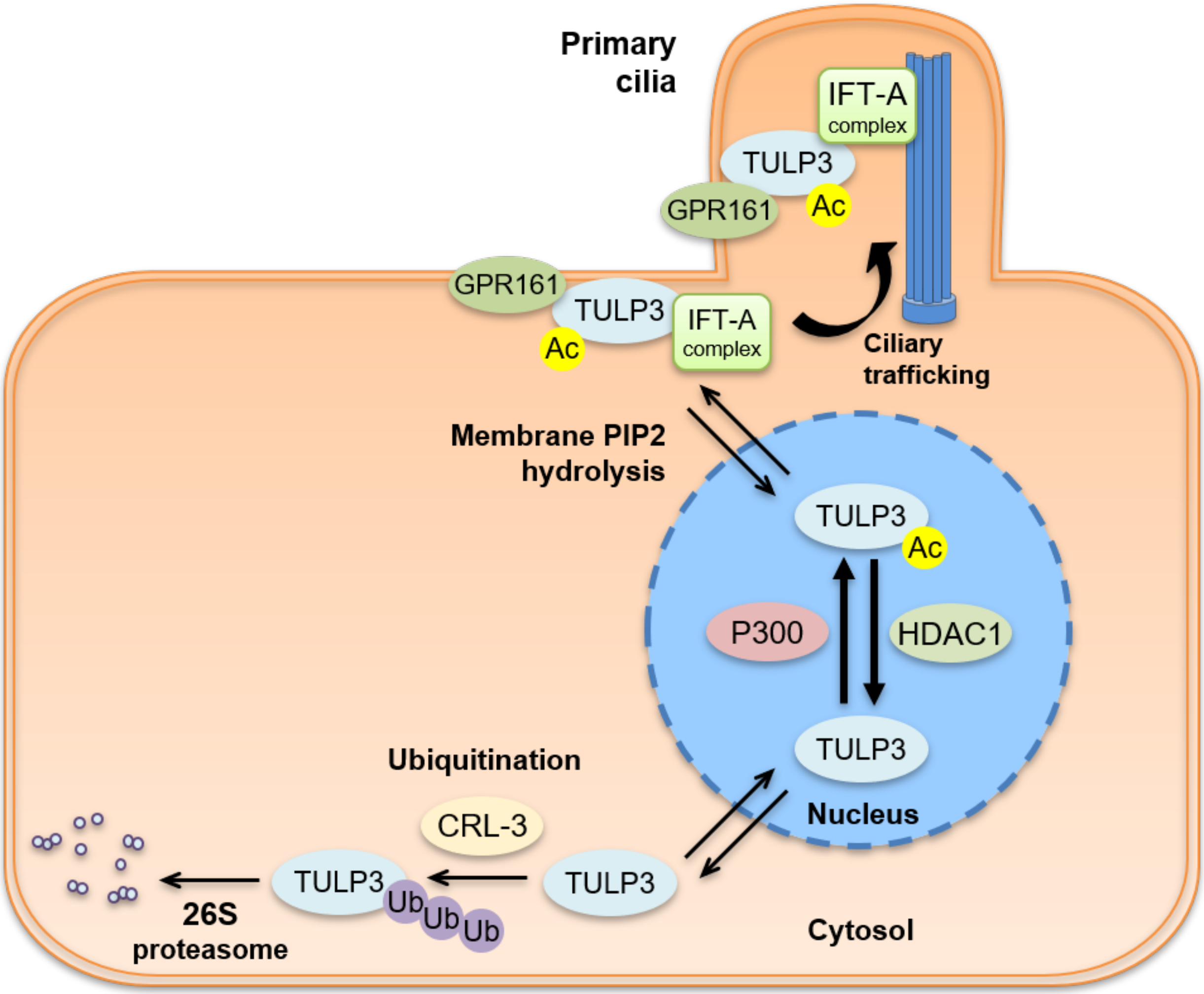

Figure S8. Model illustrating a TULP3 acetylation/ubiquitination pathway that spans both cytoplasmic and nuclear compartments. (A) Schematic outlining the involvement of a conserved TULP3 acetylation switch in assisting crosstalk between the nucleus, cytoplasm, and primary cilia.
