# Supplementary material for: A conserved acetylation switch enables pharmacological control of tubby-like protein stability
Source: J Biol Chem. 2020 Nov 23;296:100073. doi: 10.1074/jbc.RA120.015839 (PMC7948452; doi:10.1074/jbc.RA120.015839)
Supplement: Supplemental File 3 [file mmc4.pdf]

Figure 2B acetylated peptide annotated mass-labeled MS/MS spectra

<u>MOCK</u>

Lysine 37

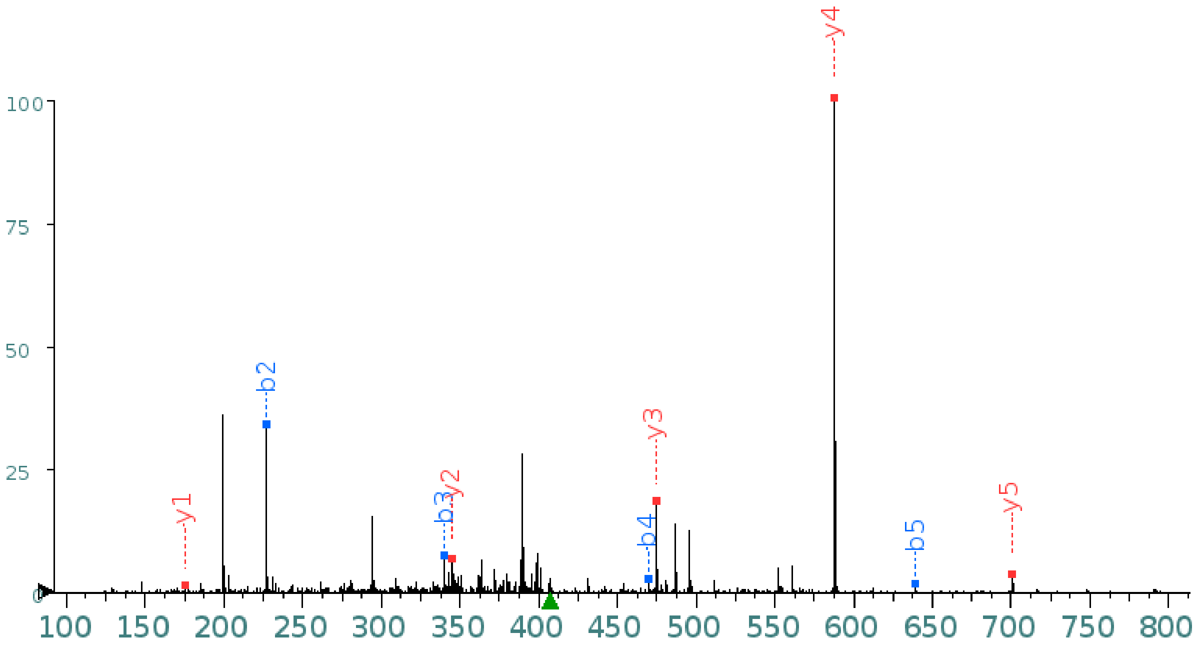

Sequence

LLLEK@R

Predicted Fragmentation Pattern

Seq	#	b: Δ Error	b	y	y: Δ Error	+1
L	1	---	114.091	---	---	6
L	2	-11.504	227.175	700.435	156.099	5
L	3	79.526	340.259	587.351	216.199	4
E	4	288.188	469.302	474.267	-6.078	3
K@	5	73.597	639.408	345.224	-103.683	2
R	6	---	---	175.119	695.744	1

Lysine 268

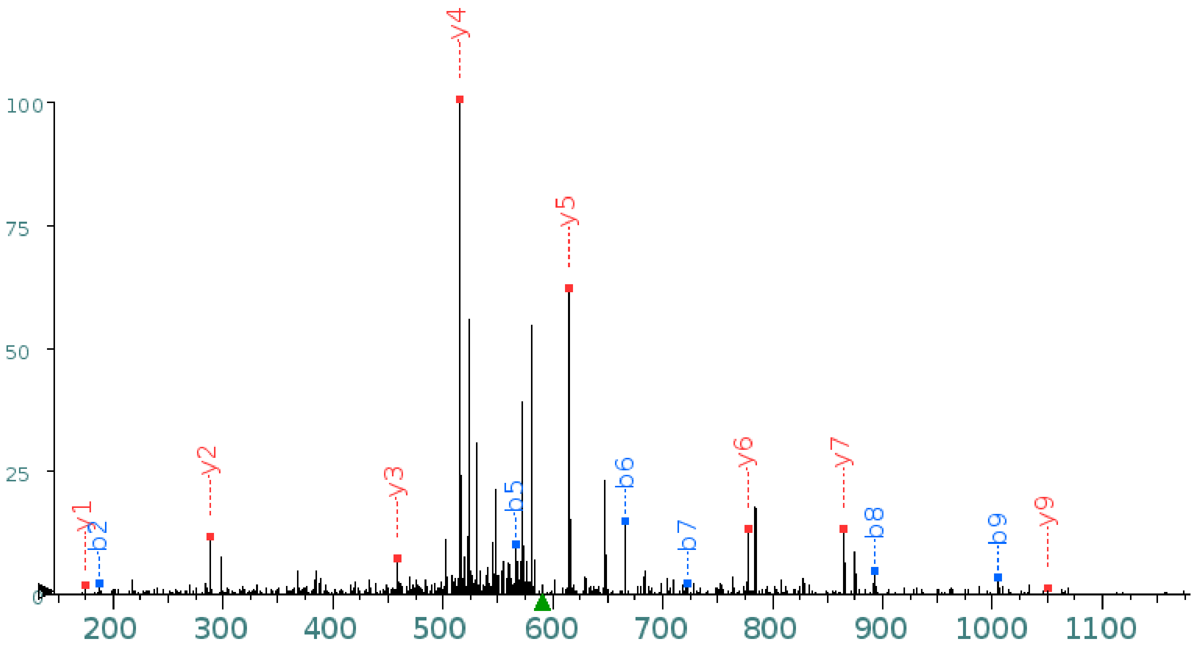

Sequence

EGESYVGK@LR

Predicted Fragmentation Pattern

Seq	#	b: Δ Error	b	y	y: Δ Error	+1
E	1	---	130.050	---	---	10
G	2	-217.983	187.071	1050.558	-297.990	9
E	3	---	316.114	993.536	---	8
S	4	---	403.146	864.494	204.699	7
Y	5	-31.466	566.209	777.462	236.849	6
V	6	189.527	665.278	614.398	151.796	5
G	7	190.899	722.299	515.330	173.974	4
K@	8	510.683	892.405	458.309	-10.280	3
L	9	140.462	1005.489	288.203	423.965	2
R	10	---	---	175.119	902.443	1

HA-p300

Lysine 37

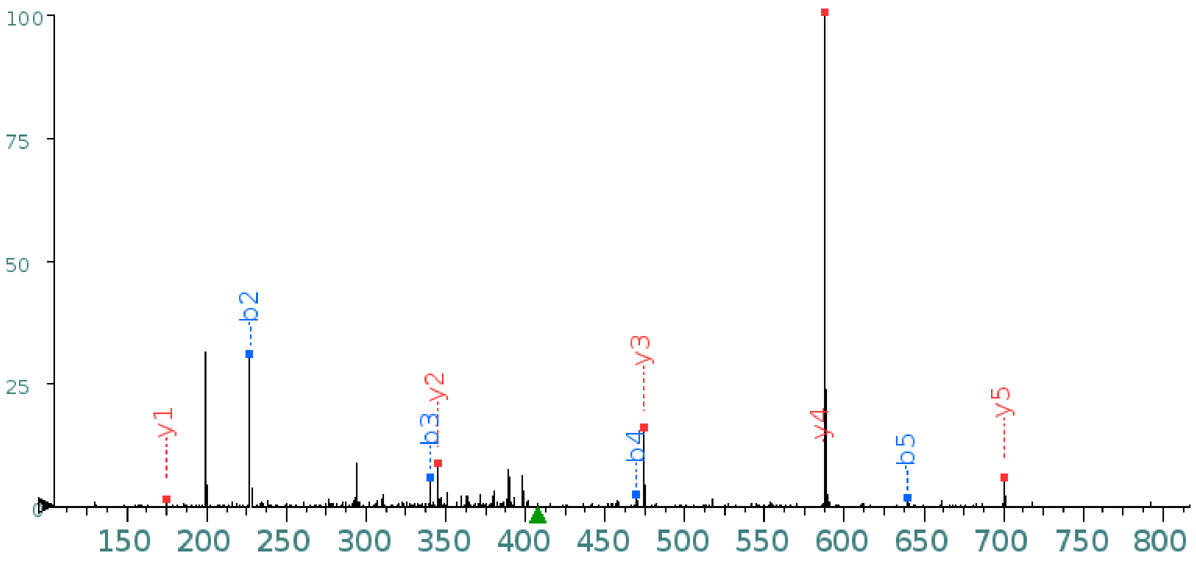

Sequence

LLLEK@R

Predicted Fragmentation Pattern

Seq	#	b: Δ Error	b	y	y: Δ Error	+1
L	1	---	114.091	---	---	6
L	2	-256.731	227.175	700.435	424.592	5
L	3	80.692	340.259	587.351	234.688	4
E	4	514.887	469.302	474.267	178.115	3
K@	5	245.554	639.408	345.224	685.881	2
R	6	---	---	175.119	668.769	1

Lysine 268

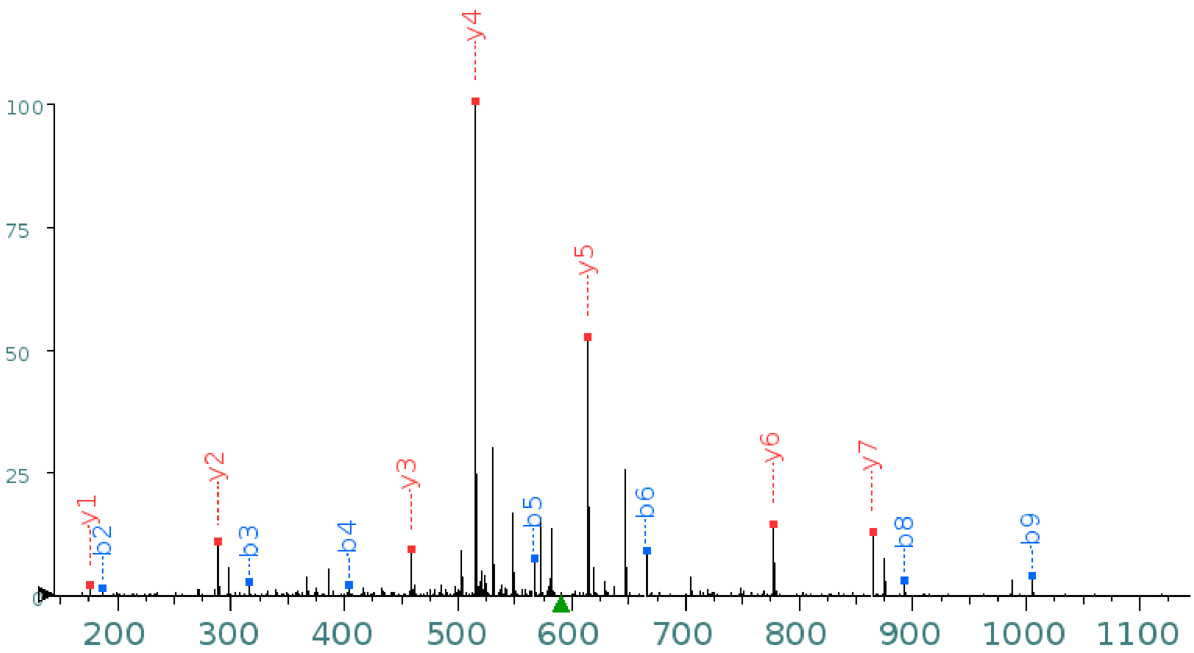

Sequence

EGESYVGK@LR

Predicted Fragmentation Pattern

Seq	#	b: Δ Error	b	y	y: Δ Error	+1
E	1	---	130.050	---	---	10
G	2	1440.928	187.071	1050.558	---	9
E	3	1105.401	316.114	993.536	---	8
S	4	841.617	403.146	864.494	107.228	7
Y	5	566.913	566.209	777.462	190.237	6
V	6	485.384	665.278	614.398	243.551	5
G	7	---	722.299	515.330	292.240	4
K@	8	289.605	892.405	458.309	756.235	3
L	9	306.468	1005.489	288.203	168.712	2
R	10	---	---	175.119	1124.706	1

Lysine 316

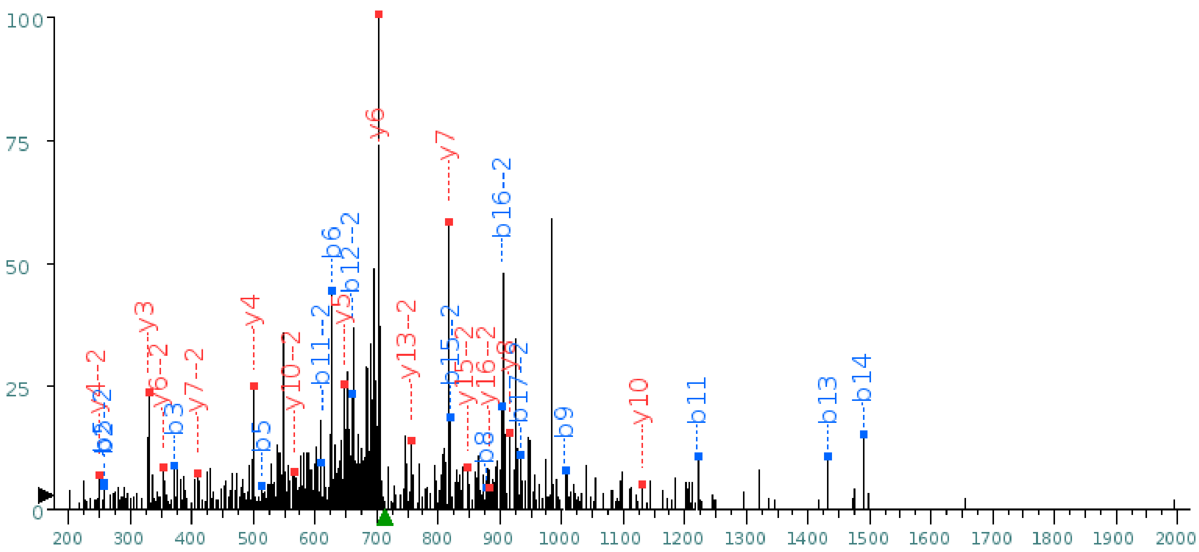

Sequence

QELAAISYETNVLGFK@GPR

Predicted Fragmentation Pattern

Seq	#	b: Δ Error	b	y	y: Δ Error	+1
Q	1	---	129.066	---	---	19
E	2	617.685	**258.108**	2007.055	---	18
L	3	418.694	**371.193**	1878.012	---	17
A	4	---	442.230	1764.928	---	16
A	5	794.050	**513.267**	1693.891	---	15
I	6	433.684	**626.351**	1622.854	---	14
S	7	---	713.383	1509.770	---	13
Y	8	343.700	**876.446**	1422.738	---	12
E	9	200.597	**1005.489**	1259.674	---	11
T	10	---	1106.536	**1130.632**	229.847	10
N	11	254.551	**1220.579**	1029.584	---	9
V	12	---	1319.648	**915.541**	-100.994	8
L	13	304.058	**1432.732**	**816.473**	427.049	7
G	14	192.929	**1489.753**	**703.389**	219.871	6
F	15	---	1636.822	**646.367**	767.872	5
K@	16	---	1806.927	**499.299**	599.355	4
G	17	---	1863.949	**329.193**	561.275	3
P	18	---	1961.001	272.172	---	2
R	19	---	---	175.119	---	1

Seq	#	b: Δ Error	b (+2)	y (+2)	y: Δ Error	+1
Q	1	---	65.037	---	---	19
E	2	---	129.558	1004.031	---	18
L	3	---	186.100	939.510	---	17
A	4	---	221.618	**882.968**	-228.583	16
A	5	364.287	**257.137**	**847.449**	170.158	15
I	6	---	313.679	811.930	---	14
S	7	---	357.195	**755.388**	284.146	13
Y	8	---	438.727	711.872	---	12
E	9	---	503.248	630.341	---	11
T	10	---	553.772	**565.819**	131.127	10
N	11	-603.198	**610.793**	515.296	---	9
V	12	448.448	**660.328**	458.274	---	8
L	13	---	716.870	**408.740**	-57.277	7
G	14	---	745.380	**352.198**	285.704	6
F	15	-537.669	**818.914**	323.687	---	5
K@	16	-15.703	**903.967**	**250.153**	222.929	4
G	17	60.058	**932.478**	165.100	---	3
P	18	---	981.004	136.589	---	2
R	19	---	---	88.063	---	1

Lysine 320

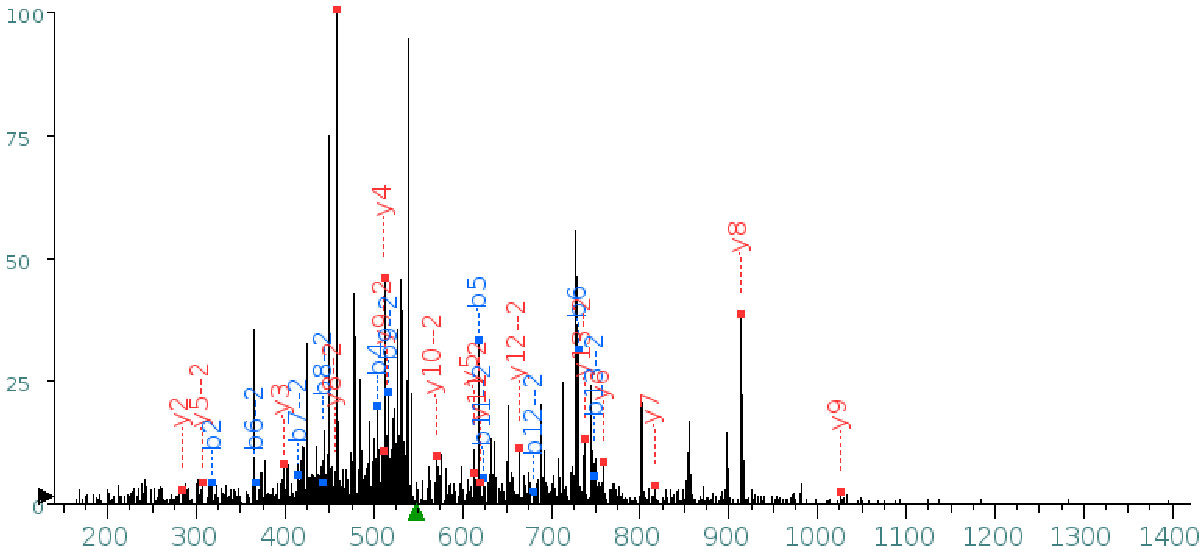

Sequence

K@M*SVIIPGM*TLNHK

Predicted Fragmentation Pattern

+1

Seq	#	b: Δ Error	b	y	y: Δ Error	+1
K@	1	---	171.113	---	---	14
M*	2	214.215	318.148	1472.760	---	13
S	3	---	405.180	1325.725	---	12
V	4	-4.697	504.249	1238.693	---	11
I	5	271.509	617.333	1139.624	---	10
I	6	239.314	730.417	1026.540	270.895	9
P	7	---	827.470	913.456	260.367	8
G	8	---	884.491	816.403	346.335	7
M*	9	---	1031.526	759.382	-324.673	6
T	10	---	1132.574	612.346	104.927	5
L	11	---	1245.658	511.299	-568.063	4
N	12	---	1359.701	398.215	818.763	3
H	13	---	1496.760	284.172	669.587	2
K	14	---	---	147.113	---	1

+2

Seq	#	b: Δ Error	b	y	y: Δ Error	+1
K@	1	---	86.060	---	---	14
M*	2	---	159.578	736.884	-17.260	13
S	3	---	203.094	663.366	29.590	12
V	4	---	252.628	619.850	-493.469	11
I	5	---	309.170	570.316	529.194	10
I	6	962.333	365.712	513.774	-937.351	9
P	7	617.273	414.238	457.232	611.635	8
G	8	-740.813	442.749	408.705	---	7
M*	9	274.574	516.267	380.195	---	6
T	10	---	566.791	306.677	-1513.095	5
L	11	591.746	623.333	256.153	---	4
N	12	212.949	680.354	199.611	---	3
H	13	-259.436	748.884	142.589	---	2
K	14	---	---	74.060	---	1

Lysine 389

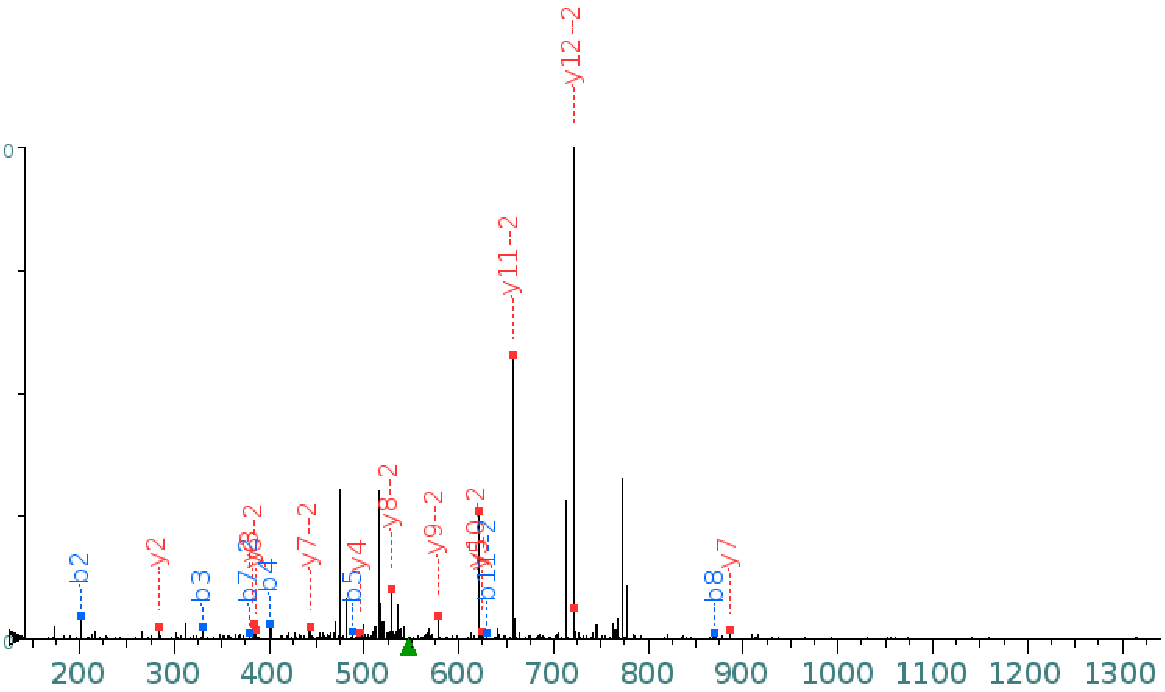

Sequence

VTQASVK@NFQIVHK

Predicted Fragmentation Pattern

			+1			
Seq	#	b: Δ Error	b	y	y: Δ Error	+1
V	1	---	100.076	---	---	14
T	2	894.055	**201.123**	1541.843	---	13
Q	3	692.877	**329.182**	1440.796	---	12
A	4	539.106	**400.219**	1312.737	---	11
S	5	673.990	**487.251**	1241.700	---	10
V	6	---	586.320	1154.668	---	9
K@	7	---	756.425	1055.600	---	8
N	8	-425.726	**870.468**	**885.494**	396.966	7
F	9	---	1017.536	771.451	---	6
Q	10	---	1145.595	**624.383**	534.682	5
I	11	---	1258.679	**496.324**	515.415	4
V	12	---	1357.747	**383.240**	953.176	3
H	13	---	1494.806	**284.172**	780.683	2
K	14	---	---	147.113	---	1

+2

Seq	#	b: Δ Error	b	y	y: Δ Error	+1
V	1	---	50.541	---	---	14
T	2	---	101.065	771.425	---	13
Q	3	---	165.095	720.902	-109.110	12
A	4	---	200.613	656.872	714.796	11
S	5	---	244.129	621.354	626.047	10
V	6	---	293.663	577.838	6.622	9
K@	7	609.801	378.716	528.303	-39.613	8
N	8	---	435.738	443.251	856.115	7
F	9	---	509.272	386.229	178.743	6
Q	10	---	573.301	312.695	---	5
I	11	-243.493	629.843	248.666	---	4
V	12	---	679.377	192.124	---	3
H	13	---	747.907	142.589	---	2
K	14	---	---	74.060	---	1

FLAG-PCAF

Lysine 268

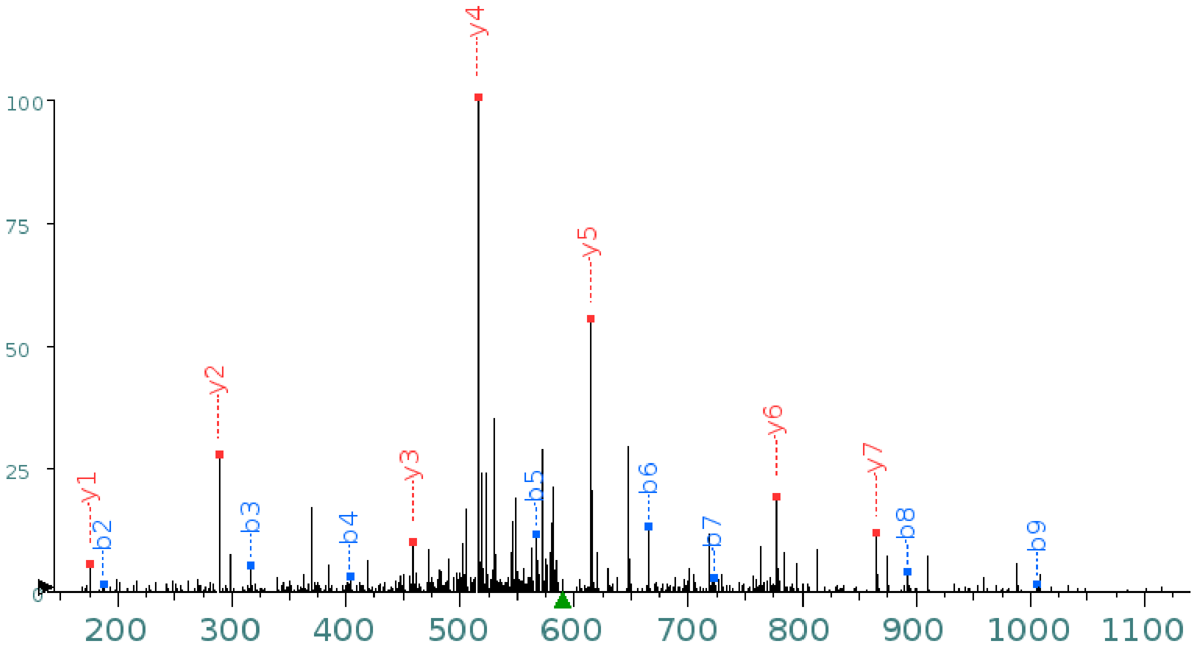

Sequence

EGESYVGK@LR

Predicted Fragmentation Pattern

Seq	#	b: Δ Error	b	y	y: Δ Error	+1
E	1	---	130.050	---	---	10
G	2	1333.965	187.071	1050.558	---	9
E	3	512.834	316.114	993.536	---	8
S	4	938.566	403.146	864.494	390.625	7
Y	5	-13.894	566.209	777.462	413.686	6
V	6	474.386	665.278	614.398	232.927	5
G	7	435.376	722.299	515.330	239.562	4
K@	8	422.537	892.405	458.309	525.739	3
L	9	364.278	1005.489	288.203	782.708	2
R	10	---	---	175.119	976.455	1

Lysine 316

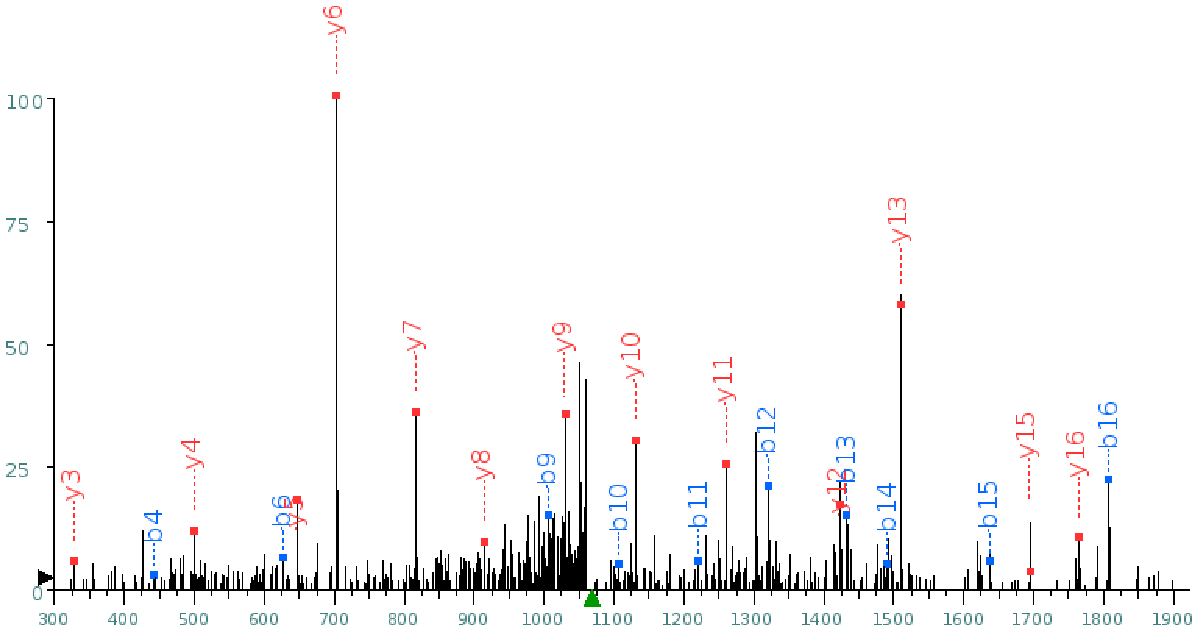

Sequence

QELAAISYETNVLGFK$^{@}$GPR

Predicted Fragmentation Pattern

Seq	#	b: Δ Error	b	y	y: Δ Error	+1
Q	1	---	129.066	---	---	19
E	2	---	258.108	2007.055	---	18
L	3	---	371.193	1878.012	---	17
A	4	486.112	442.230	1764.928	187.992	16
A	5	---	513.267	1693.891	265.964	15
I	6	554.787	626.351	1622.854	---	14
S	7	---	713.383	1509.770	148.286	13
Y	8	---	876.446	1422.738	286.030	12
E	9	272.859	1005.489	1259.674	325.058	11
T	10	206.092	1106.536	1130.632	189.701	10
N	11	259.049	1220.579	1029.584	132.529	9
V	12	349.578	1319.648	915.541	39.812	8
L	13	262.163	1432.732	816.473	341.670	7
G	14	244.856	1489.753	703.389	101.290	6
F	15	165.648	1636.822	646.367	452.773	5
K$^{@}$	16	199.931	1806.927	499.299	203.121	4
G	17	---	1863.949	329.193	204.915	3
P	18	---	1961.001	272.172	---	2
R	19	---	---	175.119	---	1

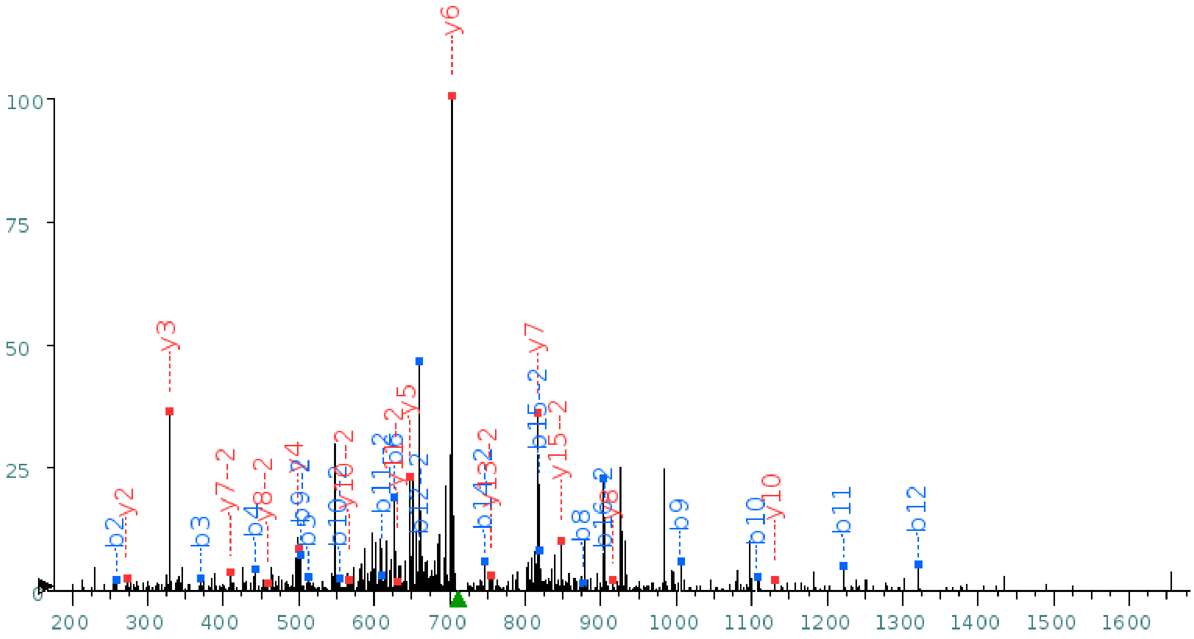

Sequence

QELAAISYETNVLGFK@GPR

Predicted Fragmentation Pattern

+1

Seq	#	b: Δ Error	b	y	y: Δ Error	+1
Q	1	---	129.066	---	---	19
E	2	994.130	**258.108**	2007.055	---	18
L	3	292.831	**371.193**	1878.012	---	17
A	4	390.688	**442.230**	1764.928	---	16
A	5	371.681	**513.267**	1693.891	---	15
I	6	258.988	**626.351**	1622.854	---	14
S	7	---	713.383	1509.770	---	13
Y	8	305.145	**876.446**	1422.738	---	12
E	9	172.017	**1005.489**	1259.674	---	11
T	10	269.715	**1106.536**	**1130.632**	277.220	10
N	11	320.220	**1220.579**	1029.584	---	9
V	12	227.176	**1319.648**	**915.541**	-443.642	8
L	13	---	1432.732	**816.473**	220.710	7
G	14	---	1489.753	**703.389**	296.799	6
F	15	---	1636.822	**646.367**	397.014	5
K@	16	---	1806.927	**499.299**	467.841	4
G	17	---	1863.949	**329.193**	156.634	3
P	18	---	1961.001	**272.172**	203.490	2
R	19	---	---	175.119	---	1

+2

Seq	#	b: Δ Error	b	y	y: Δ Error	+1
Q	1	---	65.037	---	---	19
E	2	---	129.558	1004.031	---	18
L	3	---	186.100	939.510	---	17
A	4	---	221.618	882.968	---	16
A	5	---	257.137	**847.449**	381.427	15
I	6	---	313.679	811.930	---	14
S	7	---	357.195	**755.388**	111.546	13
Y	8	---	438.727	711.872	---	12
E	9	379.899	**503.248**	**630.341**	18.962	11
T	10	210.686	**553.772**	**565.819**	36.864	10
N	11	-665.332	**610.793**	515.296	---	9
V	12	274.710	**660.328**	**458.274**	414.832	8
L	13	---	716.870	**408.740**	82.040	7
G	14	95.580	**745.380**	352.198	---	6
F	15	-26.764	**818.914**	323.687	---	5
K@	16	441.005	**903.967**	250.153	---	4
G	17	---	932.478	165.100	---	3
P	18	---	981.004	136.589	---	2
R	19	---	---	88.063	---	1

Lysine 320

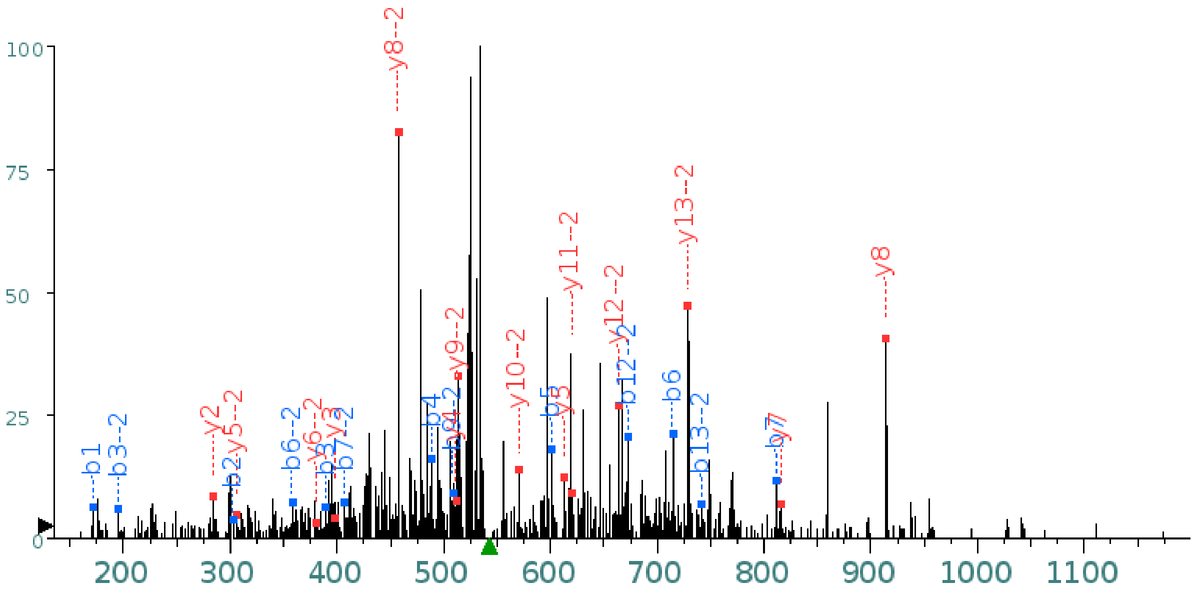

Sequence

K[@]MSVIIPGM[*]TLNHK

Predicted Fragmentation Pattern

			+1			
Seq	#	b: Δ Error	b	y	y: Δ Error	+1
K@	1	109.787	171.113	---	---	14
M	2	888.119	302.153	1456.765	---	13
S	3	580.674	389.185	1325.725	---	12
V	4	213.201	488.254	1238.693	---	11
I	5	225.639	601.338	1139.624	---	10
I	6	231.407	714.422	1026.540	---	9
P	7	151.444	811.475	913.456	133.730	8
G	8	---	868.496	816.403	379.579	7
M*	9	---	1015.531	759.382	---	6
T	10	---	1116.579	612.346	-17.263	5
L	11	---	1229.663	511.299	550.122	4
N	12	---	1343.706	398.215	106.708	3
H	13	---	1480.765	284.172	-152.068	2
K	14	---	---	147.113	---	1

Seq	#	b: Δ Error	b (+2)	y (+2)	y: Δ Error	+1
K@	1	---	86.060	---	---	14
M	2	---	151.580	728.886	162.086	13
S	3	1166.346	195.096	663.366	97.299	12
V	4	---	244.631	619.850	602.946	11
I	5	---	301.173	570.316	535.180	10
I	6	1370.293	357.715	513.774	-384.330	9
P	7	189.315	406.241	457.232	304.626	8
G	8	---	434.752	408.705	---	7
M*	9	543.022	508.269	380.195	340.672	6
T	10	---	558.793	306.677	-739.552	5
L	11	---	615.335	256.153	---	4
N	12	702.089	672.357	199.611	---	3
H	13	355.511	740.886	142.589	---	2
K	14	---	---	74.060	---	1

FLAG-GCN5

Lysine 268

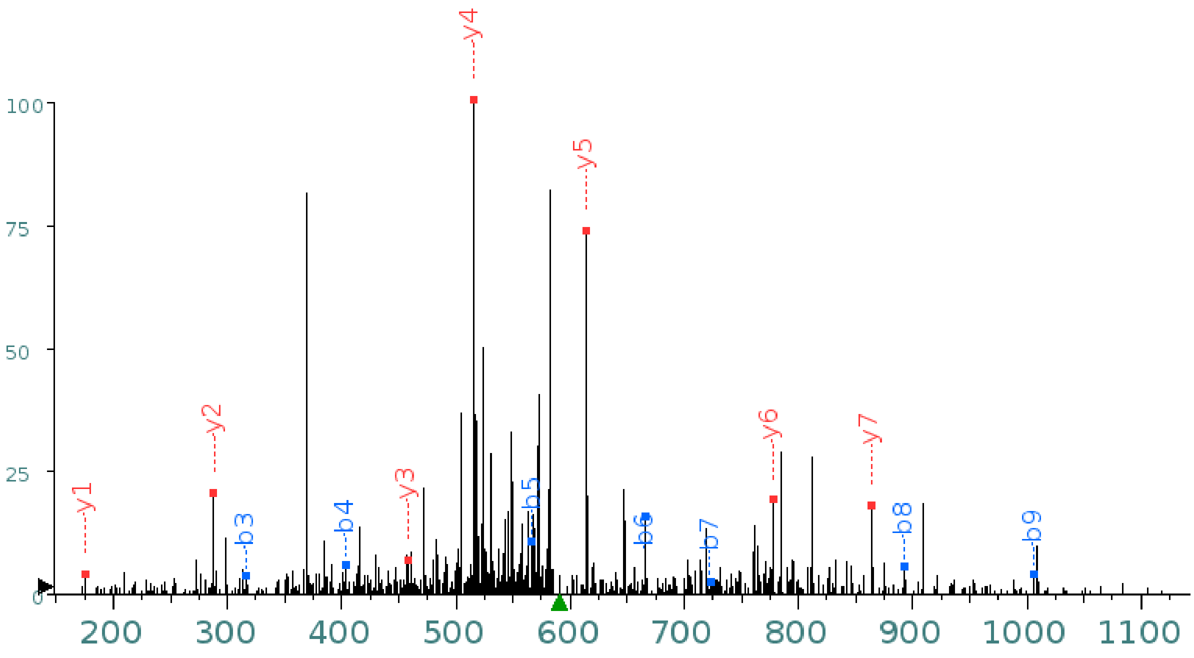

Sequence

EGESYVGK@LR

Predicted Fragmentation Pattern

Seq	#	b: Δ Error	b	y	y: Δ Error	+1
E	1	---	130.050	---	---	10
G	2	---	187.071	1050.558	---	9
E	3	397.477	316.114	993.536	---	8
S	4	784.936	403.146	864.494	207.522	7
Y	5	303.474	566.209	777.462	204.597	6
V	6	479.702	665.278	614.398	171.062	5
G	7	586.732	722.299	515.330	234.354	4
K@	8	301.567	892.405	458.309	244.823	3
L	9	177.903	1005.489	288.203	-245.558	2
R	10	---	---	175.119	970.802	1

Lysine 316

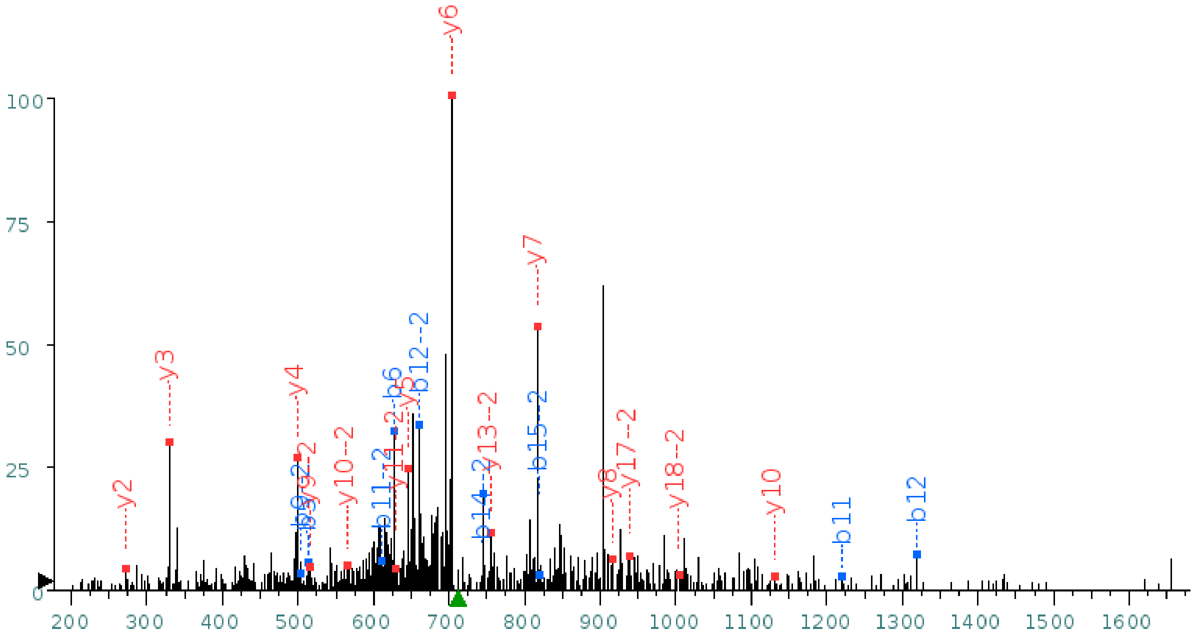

Sequence

QELAAISYETNVLGFK@GPR

Predicted Fragmentation Pattern

+1

Seq	#	b: Δ Error	b	y	y: Δ Error	+1
Q	1	---	129.066	---	---	19
E	2	---	258.108	2007.055	---	18
L	3	---	371.193	1878.012	---	17
A	4	---	442.230	1764.928	---	16
A	5	445.229	**513.267**	1693.891	---	15
I	6	419.275	**626.351**	1622.854	---	14
S	7	---	713.383	1509.770	---	13
Y	8	---	876.446	1422.738	---	12
E	9	---	1005.489	1259.674	---	11
T	10	---	1106.536	**1130.632**	407.878	10
N	11	213.266	**1220.579**	1029.584	---	9
V	12	314.635	**1319.648**	**915.541**	343.092	8
L	13	---	1432.732	**816.473**	264.346	7
G	14	---	1489.753	**703.389**	231.493	6
F	15	---	1636.822	**646.367**	550.125	5
K@	16	---	1806.927	**499.299**	342.278	4
G	17	---	1863.949	**329.193**	699.878	3
P	18	---	1961.001	**272.172**	509.600	2
R	19	---	---	175.119	---	1

+2

Seq	#	b: Δ Error	b	y	y: Δ Error	+1
Q	1	---	65.037	---	---	19
E	2	---	129.558	**1004.031**	358.556	18
L	3	---	186.100	**939.510**	194.032	17
A	4	---	221.618	882.968	---	16
A	5	---	257.137	847.449	---	15
I	6	---	313.679	811.930	---	14
S	7	---	357.195	**755.388**	316.366	13
Y	8	---	438.727	711.872	---	12
E	9	355.418	**503.248**	**630.341**	106.872	11
T	10	---	553.772	**565.819**	-817.380	10
N	11	-510.262	**610.793**	**515.296**	265.765	9
V	12	221.033	**660.328**	458.274	---	8
L	13	---	716.870	408.740	---	7
G	14	582.790	**745.380**	352.198	---	6
F	15	84.655	**818.914**	323.687	---	5
K@	16	---	903.967	250.153	---	4
G	17	---	932.478	165.100	---	3
P	18	---	981.004	136.589	---	2
R	19	---	---	88.063	---	1

Figure 5E

FLAG-TULP3 (rep1)

K232

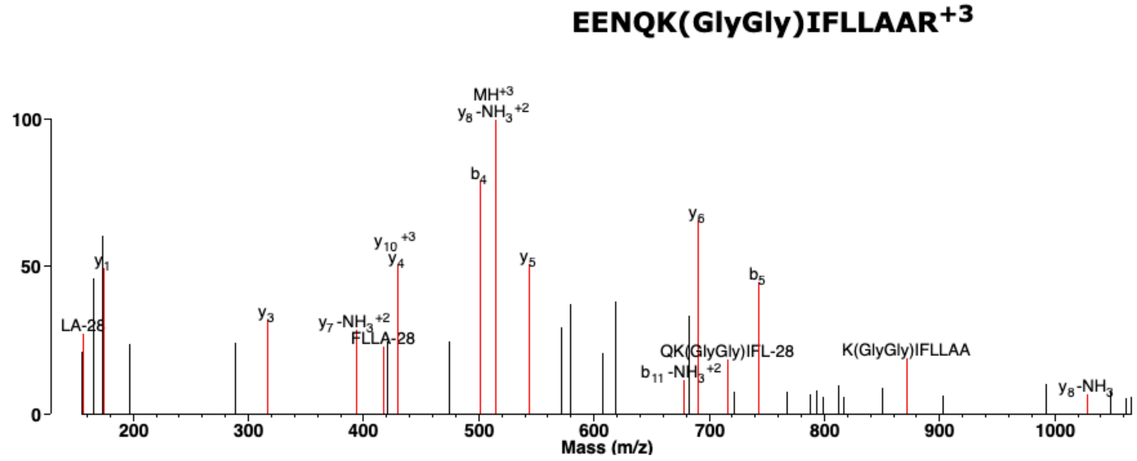

b	b^{+2}				y	y^{+2}
---	---	1	**E**	12	---	---
259.0925	---	2	**E**	11	*1416.7958*	708.9015
373.1354	---	3	**N**	10	*1287.7532*	644.3802
501.1940	---	4	**Q**	9	*1173.7103*	587.3588
743.3319	372.1696	5	**K(GlyGly)**	8	1045.6517	523.3295
856.4159	428.7116	6	**I**	7	803.5138	402.2605
1003.4843	502.2458	7	**F**	6	690.4297	345.7185
1116.5684	558.7878	8	**L**	5	543.3613	272.1843
1229.6525	615.3299	9	**L**	4	430.2772	215.6423
1300.6896	650.8484	10	**A**	3	317.1932	159.1002
1371.7267	686.3670	11	**A**	2	246.1561	*123.5817*
---	---	12	**R**	1	175.1190	*88.0631*

K316

QELAAISYETNVLGFK(GlyGly)GPR^{+3}

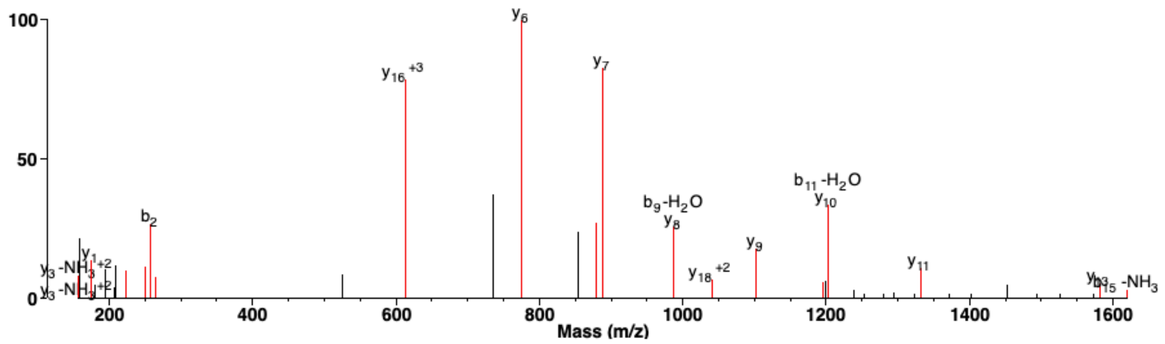

b	b^{+2}				y	y^{+2}
---	---	1	Q	19	---	---
258.1084	---	2	E	18	*2079.0869*	1040.0471
371.1925	---	3	L	17	*1950.0443*	975.5258
442.2296	---	4	A	16	*1836.9603*	918.9838
513.2667	---	5	A	15	*1765.9232*	883.4652
626.3508	---	6	I	14	*1694.8860*	847.9467
713.3828	---	7	S	13	1581.8020	791.4046
876.4462	---	8	Y	12	1494.7700	747.8886
1005.4888	---	9	E	11	1331.7066	666.3570
1106.5364	---	10	T	10	1202.6640	601.8357
1220.5794	---	11	N	9	1101.6164	551.3118
1319.6478	---	12	V	8	987.5734	494.2904
1432.7318	---	13	L	7	888.5050	444.7561
1489.7533	---	14	G	6	775.4209	388.2141
1636.8217	---	15	F	5	718.3995	359.7034
1878.9596	939.9834	16	K(GlyGly)	4	571.3311	286.1692
1935.9811	968.4942	17	G	3	329.1932	165.1002
2033.0338	1017.0206	18	P	2	272.1717	*136.5895*
---	---	19	R	1	175.1190	*88.0631*

FLAG-TULP3 (rep2)

K27

QAK(GlyGly)LDYQR^{+3}

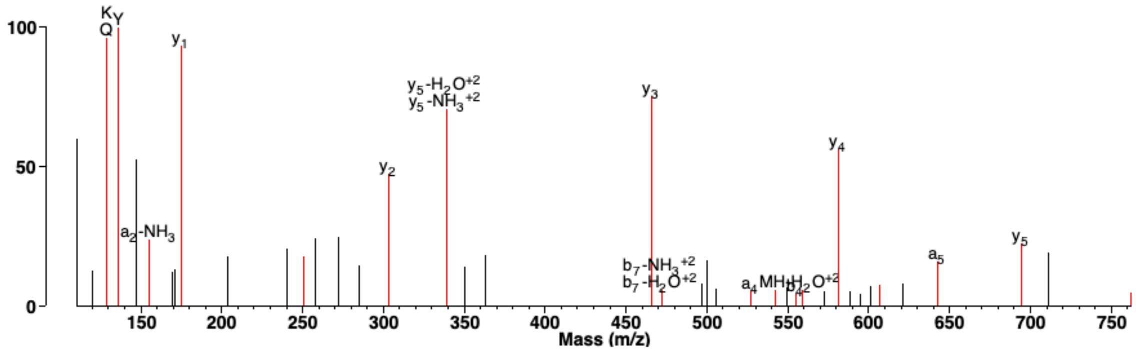

b	b^{+2}				y	y^{+2}
---	---	1	**Q**	8	---	---
200.1030	---	2	**A**	7	*1007.5269*	504.2671
442.2409	221.6241	3	**K(GlyGly)**	6	*936.4898*	468.7485
555.3249	278.1661	4	**L**	5	694.3519	347.6796
670.3519	335.6796	5	**D**	4	581.2678	291.1375
833.4152	417.2112	6	**Y**	3	466.2409	233.6241
961.4738	481.2405	7	**Q**	2	303.1775	152.0924
---	---	8	**R**	1	175.1190	*88.0631*

K232

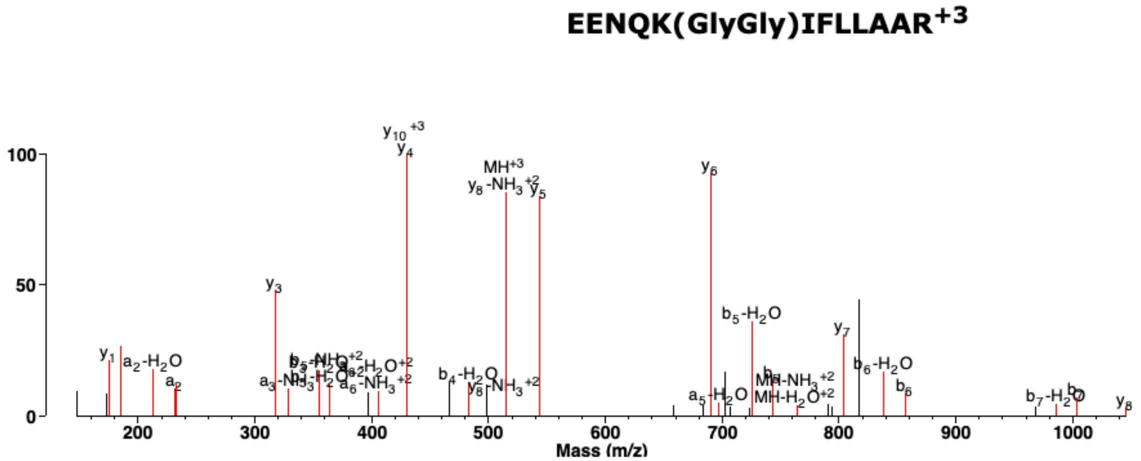

b	b+2				y	y+2
---	---	1	E	12	---	---
259.0925	---	2	E	11	*1416.7958*	708.9015
373.1354	---	3	N	10	*1287.7532*	644.3802
501.1940	---	4	Q	9	*1173.7103*	587.3588
743.3319	372.1696	5	K(GlyGly)	8	1045.6517	523.3295
856.4159	428.7116	6	I	7	803.5138	402.2605
1003.4843	502.2458	7	F	6	690.4297	345.7185
1116.5684	558.7878	8	L	5	543.3613	272.1843
1229.6525	615.3299	9	L	4	430.2772	215.6423
1300.6896	650.8484	10	A	3	317.1932	159.1002
1371.7267	686.3670	11	A	2	246.1561	*123.5817*
---	---	12	R	1	175.1190	*88.0631*

K245

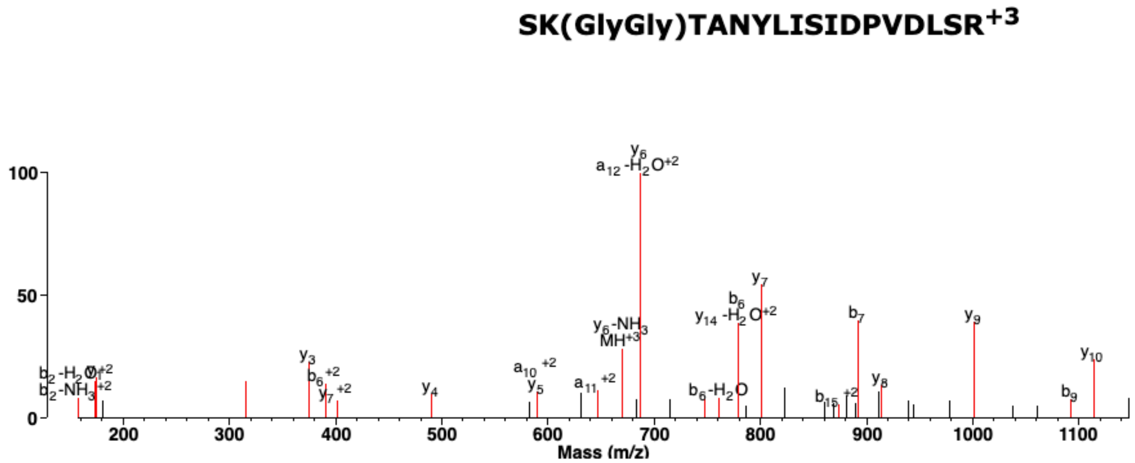

b	b^{+2}				y	y^{+2}
---	---	1	S	17	---	---
330.1772	165.5922	2	K(GlyGly)	16	1919.0233	960.0153
431.2249	216.1161	3	T	15	1676.8854	838.9463
502.2620	251.6346	4	A	14	1575.8377	788.4225
616.3049	308.6561	5	N	13	1504.8006	752.9039
779.3682	390.1878	6	Y	12	1390.7577	695.8825
892.4523	446.7298	7	L	11	1227.6943	614.3508
1005.5364	503.2718	8	I	10	1114.6103	557.8088
1092.5684	546.7878	9	S	9	1001.5262	501.2667
1205.6525	603.3299	10	I	8	914.4942	457.7507
1320.6794	660.8433	11	D	7	801.4101	401.2087
1417.7322	709.3697	12	P	6	686.3832	343.6952
1516.8006	758.9039	13	V	5	589.3304	295.1688
1631.8275	816.4174	14	D	4	490.2620	245.6346
1744.9116	872.9594	15	L	3	375.2350	188.1212
1831.9436	916.4754	16	S	2	262.1510	131.5791
---	---	17	R	1	175.1190	88.0631

K277

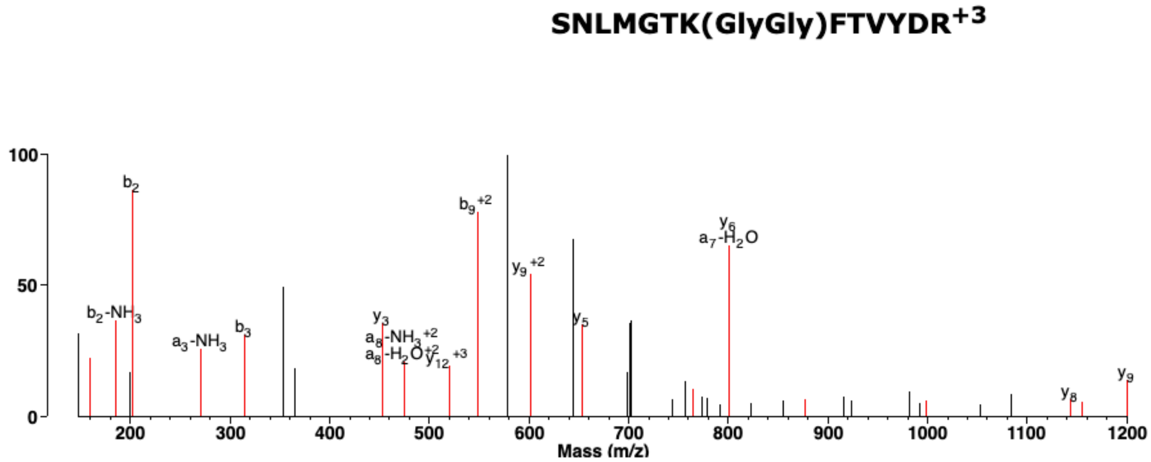

b	b+2				y	y+2
---	---	1	S	13	---	---
202.0822	---	2	N	12	1558.7682	779.8878
315.1663	---	3	L	11	1444.7253	722.8663
446.2068	---	4	M	10	1331.6412	666.3243
503.2282	---	5	G	9	1200.6008	600.8040
604.2759	---	6	T	8	1143.5793	572.2933
846.4138	423.7105	7	K(GlyGly)	7	1042.5316	521.7694
993.4822	497.2448	8	F	6	800.3937	400.7005
1094.5299	547.7686	9	T	5	653.3253	327.1663
1193.5983	597.3028	10	V	4	552.2776	276.6425
1356.6616	678.8345	11	Y	3	453.2092	227.1083
1471.6886	736.3479	12	D	2	290.1459	145.5766
---	---	13	R	1	175.1190	88.0631

TULP only

K27

QAK(Acetyl)LDYQR^{+2}

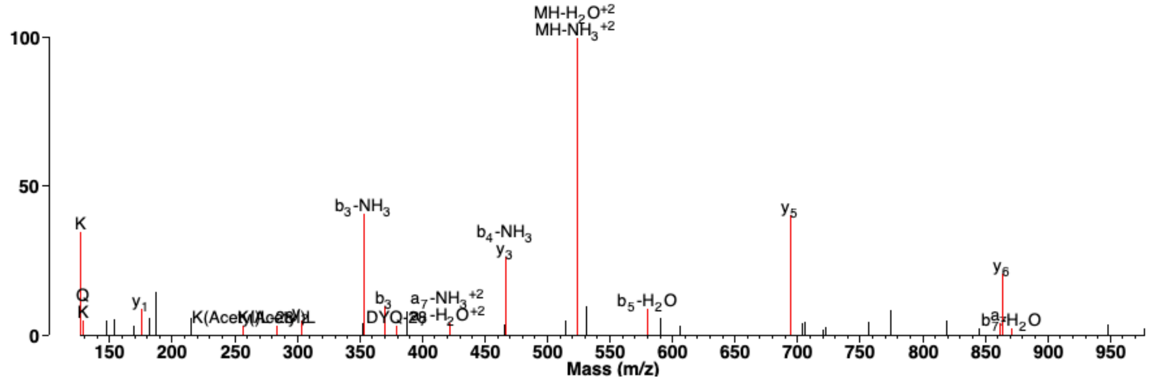

b	b^{+2}				y	y^{+2}
---	---	1	**Q**	8	---	---
200.1030	---	2	**A**	7	935.4945	468.2509
370.2085	185.6079	3	**K(Acetyl)**	6	864.4574	432.7323
483.2926	242.1499	4	**L**	5	694.3519	347.6796
598.3195	299.6634	5	**D**	4	581.2678	291.1375
761.3828	381.1951	6	**Y**	3	466.2409	233.6241
889.4414	445.2243	7	**Q**	2	303.1775	152.0924
---	---	8	**R**	1	175.1190	*88.0631*

K364

TM(Oxidation)ENLVELHN(Deamidated)K(Acetyl)APVWNSDTQSYVLNFR^{+4}

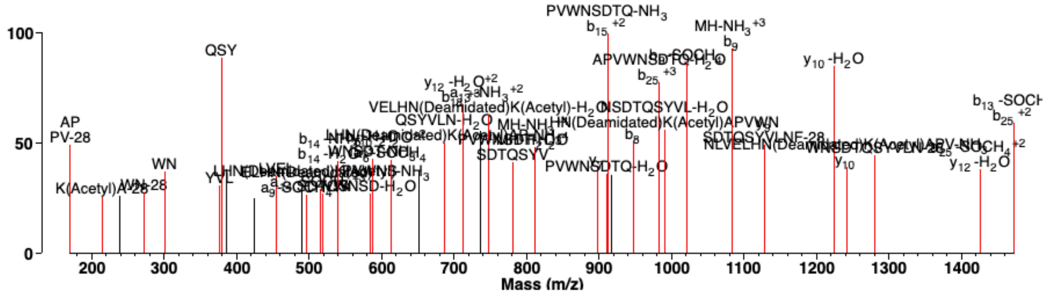

b	b^{+2}				y	y^{+2}
---	---	1	**T**	27	---	---
249.0904	---	2	**M(Oxidation)**	26	3163.5153	1582.2613
378.1329	---	3	**E**	25	3016.4799	1508.7436
492.1759	---	4	**N**	24	2887.4373	1444.2223
605.2599	---	5	**L**	23	2773.3944	1387.2008
704.3284	---	6	**V**	22	2660.3103	1330.6588
833.3709	---	7	**E**	21	2561.2419	1281.1246
946.4550	---	8	**L**	20	2432.1993	1216.6033
1083.5139	542.2606	9	**H**	19	2319.1153	1160.0613
1198.5409	599.7741	10	**N(Deamidated)**	18	2182.0564	1091.5318
1368.6464	684.8268	11	**K(Acetyl)**	17	2067.0294	1034.0183
1439.6835	720.3454	12	**A**	16	1896.9239	948.9656
1536.7363	768.8718	13	**P**	15	1825.8868	913.4470
1635.8047	818.4060	14	**V**	14	1728.8340	864.9206
1821.8840	911.4456	15	**W**	13	1629.7656	815.3864
1935.9269	968.4671	16	**N**	12	1443.6863	722.3468
2022.9590	1011.9831	17	**S**	11	1329.6434	665.3253
2137.9859	1069.4966	18	**D**	10	1242.6113	621.8093
2239.0336	1120.0204	19	**T**	9	1127.5844	564.2958
2367.0922	1184.0497	20	**Q**	8	1026.5367	513.7720
2454.1242	1227.5657	21	**S**	7	898.4781	449.7427
2617.1875	1309.0974	22	**Y**	6	811.4461	406.2267
2716.2559	1358.6316	23	**V**	5	648.3828	324.6950
2829.3400	1415.1736	24	**L**	4	549.3144	275.1608
2943.3829	1472.1951	25	**N**	3	436.2303	218.6188
3090.4513	1545.7293	26	**F**	2	322.1874	161.5973
---	---	27	**R**	1	175.1190	88.0631

TULP3 + p300

K37

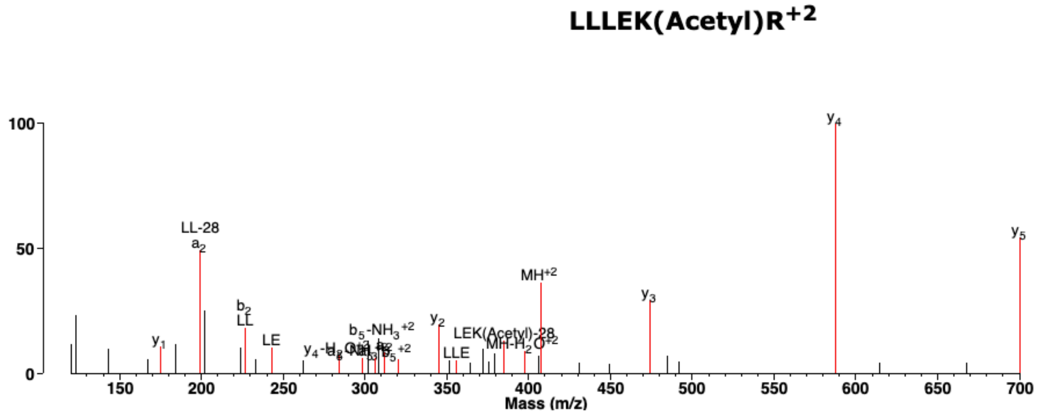

b	b^{+2}				y	y^{+2}
---	---	1	**L**	6	---	---
227.1754	---	2	**L**	5	700.4352	350.7212
340.2595	---	3	**L**	4	587.3511	294.1792
469.3021	---	4	**E**	3	474.2671	237.6372
639.4076	320.2074	5	**K(Acetyl)**	2	345.2245	173.1159
---	---	6	**R**	1	175.1190	88.0631

TULP3 + p300 + acetyl coA

K37

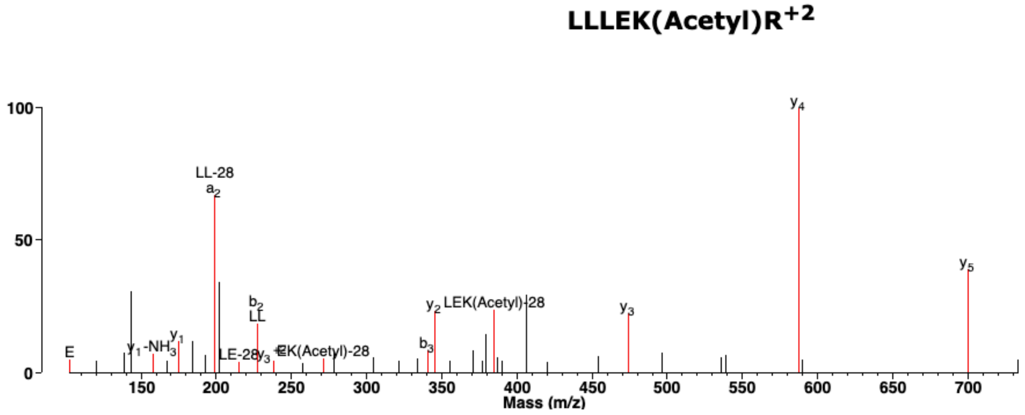

b	b^{+2}				y	y^{+2}
---	---	1	**L**	6	---	---
227.1754	---	2	**L**	5	700.4352	350.7212
340.2595	---	3	**L**	4	587.3511	294.1792
469.3021	---	4	**E**	3	474.2671	237.6372
639.4076	320.2074	5	**K(Acetyl)**	2	345.2245	173.1159
---	---	6	**R**	1	175.1190	88.0631

K268

EGESYVGK(Acetyl)LR^{+2}

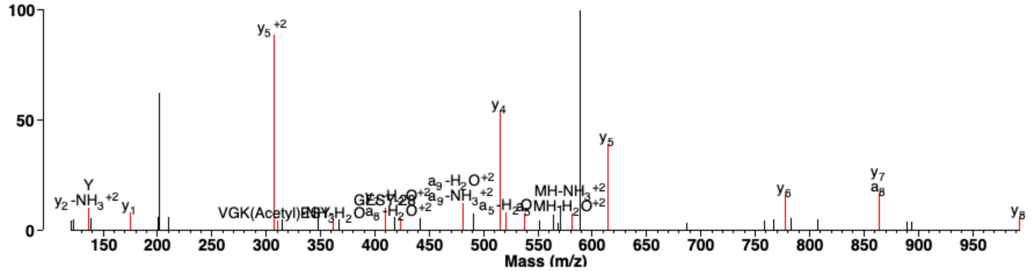

b	b^{+2}				y	y^{+2}
---	---	1	**E**	10	---	---
187.0713	---	2	**G**	9	*1050.5578*	525.7826
316.1139	---	3	**E**	8	993.5364	497.2718
403.1460	---	4	**S**	7	864.4938	432.7505
566.2093	---	5	**Y**	6	777.4618	389.2345
665.2777	---	6	**V**	5	614.3984	307.7028
722.2992	---	7	**G**	4	515.3300	258.1686
892.4047	446.7060	8	**K(Acetyl)**	3	458.3085	229.6579
1005.4888	503.2480	9	**L**	2	288.2030	144.6051
---	---	10	**R**	1	175.1190	*88.0631*

K277

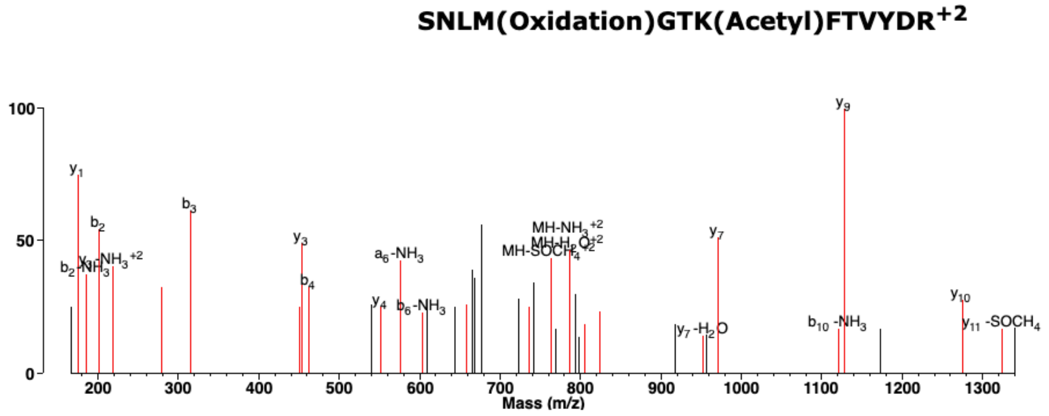

b	b^{+2}				y	y^{+2}
---	---	1	**S**	13	---	---
202.0822	---	2	**N**	12	*1502.7308*	751.8690
315.1663	---	3	**L**	11	*1388.6879*	694.8476
462.2017	---	4	**M(Oxidation)**	10	1275.6038	638.3055
519.2232	---	5	**G**	9	1128.5684	564.7878
620.2708	---	6	**T**	8	1071.5469	536.2771
790.3764	395.6918	7	**K(Acetyl)**	7	970.4993	485.7533
937.4448	469.2260	8	**F**	6	800.3937	400.7005
1038.4925	519.7499	9	**T**	5	653.3253	327.1663
1137.5609	569.2841	10	**V**	4	552.2776	276.6425
1300.6242	650.8157	11	**Y**	3	453.2092	227.1083
1415.6511	708.3292	12	**D**	2	290.1459	*145.5766*
---	---	13	**R**	1	175.1190	*88.0631*

TULP3 + p300 + acetyl coA + C646

K27

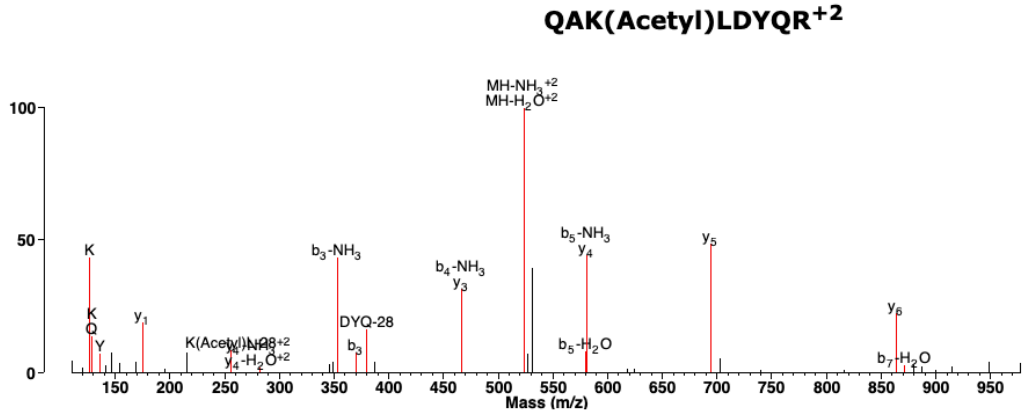

b	b^{+2}				y	y^{+2}
---	---	1	**Q**	8	---	---
200.1030	---	2	**A**	7	935.4945	468.2509
370.2085	185.6079	3	**K(Acetyl)**	6	864.4574	432.7323
483.2926	242.1499	4	**L**	5	694.3519	347.6796
598.3195	299.6634	5	**D**	4	581.2678	291.1375
761.3828	381.1951	6	**Y**	3	466.2409	233.6241
889.4414	445.2243	7	**Q**	2	303.1775	152.0924
---	---	8	**R**	1	175.1190	*88.0631*

K37

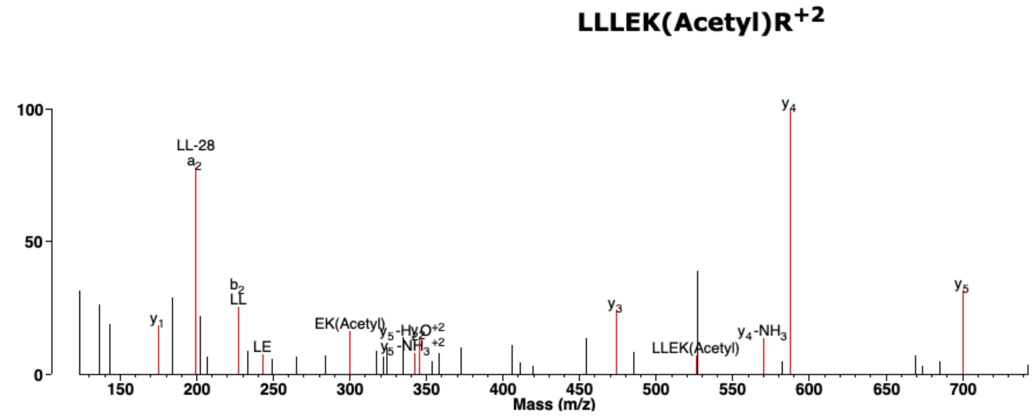

b	b^{+2}				y	y^{+2}
---	---	1	**L**	6	---	---
227.1754	---	2	**L**	5	700.4352	350.7212
340.2595	---	3	**L**	4	587.3511	294.1792
469.3021	---	4	**E**	3	474.2671	237.6372
639.4076	320.2074	5	**K(Acetyl)**	2	345.2245	173.1159
---	---	6	**R**	1	175.1190	*88.0631*
